# Neoadjuvant PD-1 and LAG-3-targeting bispecific antibody and other immune checkpoint inhibitor combinations in resectable melanoma: the randomized phase 1b/2 Morpheus-Melanoma trial

**DOI:** 10.1038/s41591-025-03967-2

**Published:** 2025-09-24

**Authors:** Georgina V. Long, Nitya Nair, Daniel Marbach, Richard A. Scolyer, Sabine Wilson, Denise Cotting, Nicolas Staedler, Rodabe N. Amaria, Paolo Antonio Ascierto, Ahmad A. Tarhini, Caroline Robert, Omid Hamid, Caroline Gaudy-Marqueste, Celeste Lebbe, Eva Munoz-Couselo, Alexander M. Menzies, Cecile Pages, Giuseppe Curigliano, Mario Mandala, Nikki Jessop, Uwe Bader, Maurizio Perdicchio, Volker Teichgräber, Merlind Muecke, Christoph Markert, Christian Blank

**Affiliations:** 1 Melanoma Institute Australia, The University of Sydney, Sydney, New South Wales Australia; 2 Faculty of Medicine and Health, The University of Sydney, Sydney, New South Wales Australia; 3 Royal North Shore Hospital and Mater Hospitals, Sydney, New South Wales Australia; 4 Roche Pharma Research and Early Development, Roche Innovation Center Basel, F. Hoffmann-La Roche, Ltd., Basel, Switzerland; 5 Royal Prince Alfred Hospital and NSW Health Pathology, Sydney, New South Wales Australia; 6 Charles Perkins Centre, The University of Sydney, Sydney, New South Wales Australia; 7 Roche Pharma Research and Early Development, Roche Innovation Center Welwyn, Roche Products, Ltd., Welwyn Garden City, UK; 8 F. Hoffmann-La Roche, Ltd., Basel, Switzerland; 9 The University of Texas MD Anderson Cancer Center, Houston, TX USA; 10 Istituto Nazionale Tumori IRCCS Fondazione G. Pascale, Naples, Italy; 11 H. Lee Moffitt Cancer Center & Research Institute, Tampa, FL USA; 12 Gustave Roussy and Paris Saclay University, Villejuif, France; 13 The Angeles Clinic, Los Angeles, CA USA; 14 Department of Dermatology and Skin Cancer, Aix-Marseille University, APHM, CEPCM, Hôpital Timone, Marseille, France; 15 Université Paris Cité, AP-HP Dermato-oncology and CIC, Cancer Institute APHP Nord Paris Cité, INSERM U1342 - Equipe 1 - CNRS EMR8000, Saint-Louis Hospital, Paris, France; 16 Vall d’Hebron Institute of Oncology, Barcelona, Spain; 17 Vall d’Hebron Hospital, Barcelona, Spain; 18 Oncopole Claudius Regaud and Institut Universitaire du Cancer, Toulouse, France; 19 European Institute of Oncology, IRCCS, Milano, Italy; 20 Department of Oncology and Hemato-Oncology, University of Milano, Milano, Italy; 21 University of Perugia, Santa Maria Misericordia Hospital, Perugia, Italy; 22 Roche Pharma Research and Early Development, Roche Diagnostics GmbH, Penzberg, Germany; 23 Netherlands Cancer Institute (NKI), Amsterdam, The Netherlands; 24 Leiden University Medical Center (LUMC), Leiden, The Netherlands; 25 University Clinic Regensburg (UKR), Regensburg, Germany

**Keywords:** Melanoma, Tumour biomarkers, Cancer immunotherapy, Translational research

## Abstract

Patients with stage III melanoma are at high risk of relapse. The NADINA trial evaluating neoadjuvant nivolumab plus ipilimumab and the SWOG-1801 trial evaluating neoadjuvant pembrolizumab have demonstrated superior clinical outcomes with neoadjuvant versus adjuvant checkpoint inhibition. Morpheus-Melanoma was a phase 1b/2, randomized umbrella trial evaluating tobemstomig (anti-PD-1/anti-LAG-3 bispecific antibody; *n* = 40), tobemstomig plus tiragolumab (anti-TIGIT monoclonal antibody; *n* = 20) and atezolizumab (PD-L1-targeting monoclonal antibody) plus tiragolumab (*n* = 20) versus nivolumab (anti-PD-1 monoclonal antibody) plus ipilimumab (anti-CTLA-4 monoclonal antibody; *n* = 22) in stage III melanoma. The primary endpoint was pathological response by independent pathological review. Additional endpoints included safety and exploratory biomarkers. Here tobemstomig showed a similar pathological response rate (pRR) versus nivolumab plus ipilimumab (80.0% (32/40) versus 77.3% (17/22)); major pathological responses were less frequent with tobemstomig versus nivolumab plus ipilimumab treatment (62.5% (25/40) versus 72.7% (16/22)). Tobemstomig plus tiragolumab and atezolizumab plus tiragolumab showed a lower pRR versus nivolumab plus ipilimumab (60.0% (12/20) and 45.0% (9/20) versus 77.3% (17/22), respectively). Tobemstomig demonstrated improved safety versus nivolumab plus ipilimumab, with 2.5% (1/40) and 22.7% (5/22) of patients experiencing grade 3 or higher treatment-related adverse events (TRAEs), respectively, and 0% (0/40) and 13.6% (3/22) of patients discontinuing treatment due to TRAEs, respectively. Grade 3 or higher TRAEs were reported by 15% (3/20) of patients in the tobemstomig plus tiragolumab arm and by no patients in the atezolizumab plus tiragolumab arm. Baseline CD8^+^ and CD3^+^ tumor-infiltrating T cell density, IFNγ pathway and effector T cell gene expression, tumor mutational burden and pre-surgery circulating tumor DNA correlated with pathological response across treatments. In conclusion, in the Morpheus-Melanoma study, tobemstomig demonstrated a similar pathological response and improved safety profile versus nivolumab plus ipilimumab in patients with resectable stage III melanoma. ClinicalTrials.gov identifier: NCT05116202.

## Abstract (web summary)

In the randomized phase 1b/2 Morpheus-Melanoma trial evaluating various neoadjuvant immune checkpoint inhibitor regimens in patients with resectable stage III melanoma, tobemstomig, an anti-PD-1/anti-LAG-3 bispecific antibody, showed the highest pathologic response rate with a better safety profile than the standard treatment approach of ipilimumab plus nivolumab.

## Main

Current standard of care for clinically detectable, resectable stage III melanoma includes adjuvant anti-programmed cell death protein 1 (PD-1) therapy or *BRAF*-targeted therapy for patients treated with upfront resection, or neoadjuvant nivolumab plus ipilimumab followed by adjuvant therapy based on pathological response and *BRAF* status, or neoadjuvant plus adjuvant pembrolizumab^1–5^. In the phase 2 SWOG-1801 study, event-free survival (EFS) at 2 years was 72% in patients who received neoadjuvant–adjuvant pembrolizumab versus 49% in patients who received pembrolizumab adjuvant-only therapy, demonstrating the superiority of neoadjuvant checkpoint inhibitor (CPI) treatment over adjuvant CPI treatment in advanced melanoma^4^. In addition, grade 3 or higher TRAEs were similar in the neoadjuvant–adjuvant and adjuvant-only groups (12% versus 14%, respectively)^4^. In the phase 3 NADINA study, neoadjuvant nivolumab plus ipilimumab followed by surgery and response-driven adjuvant therapy resulted in a 68% reduction in the risk of disease recurrence or death versus surgery plus adjuvant nivolumab^5^. However, a substantially higher rate of grade 3 or higher TRAEs was observed with neoadjuvant nivolumab plus ipilimumab versus adjuvant nivolumab (29.7% versus 14.7%, respectively)^5^.

Pathological response to neoadjuvant immunotherapy in melanoma is associated with long-term survival outcomes^6,7^. Neoadjuvant nivolumab plus ipilimumab has demonstrated higher major pathological response (MPR) versus pembrolizumab but at the cost of higher clinically meaningful toxicity^5,8^. Therefore, there is a need for alternative strategies and novel combinations to increase MPR, reduce toxicities and enable personalized neoadjuvant treatment options.

PD-1 and lymphocyte-activation gene 3 (LAG-3) are inhibitory immune checkpoints that are often co-expressed on tumor-infiltrating lymphocytes (TILs)^9^. In phase 2/3 trials, concurrent checkpoint inhibition of PD-1 and LAG-3 led to an improved progression-free survival versus PD-1 inhibition alone in patients with previously untreated metastatic melanoma^9^ and high MPR (63%) in patients with resectable clinical stage III melanoma^10^.

Tobemstomig is a novel, Fc-silent, IgG1-based bispecific antibody that simultaneously targets PD-1 and LAG-3 (ref. ^11^). Tobemstomig is designed with 20-fold higher binding affinity to PD-1 over LAG-3, resulting in an avidity-driven selectivity gain to PD-1 and LAG-3 co-expressing activated effector T (T_eff_) cells in the tumor over regulatory T (T_reg_) cells that constitutively express LAG-3 (ref. ^11^). Treatment with tobemstomig may reinvigorate TILs, independent of T_reg_ cells, and potentially delay, prevent or overcome development of LAG-3-mediated adaptive resistance mechanisms^11^. In a phase 1 study, single-agent tobemstomig demonstrated a tolerable safety profile and encouraging antitumor activity in patients with advanced and/or metastatic solid tumors with or without prior CPI exposure (NCT04140500)^12^.

Tiragolumab is an anti-TIGIT monoclonal antibody that binds to TIGIT, an immune checkpoint molecule, and prevents its binding to the polio virus receptor (PvR) and its counterreceptor CD226 (ref. ^13^), resulting in increased PvR–CD226 binding and T cell activation. Atezolizumab is a programmed death ligand 1 (PD-L1)-targeting monoclonal antibody approved as first-line treatment for non-small cell lung cancer (NSCLC), among other solid tumor indications. Tiragolumab plus atezolizumab demonstrated increased efficacy versus atezolizumab alone in first-line NSCLC^13^.

Here we report efficacy, safety and biomarker results from Morpheus-Melanoma (NCT05116202), a phase 1b/2, open-label, multicenter, randomized umbrella study in treatment-naive patients with clinically detectable, Response Evaluation Criteria in Solid Tumors (RECIST)-measurable and resectable stage III melanoma who received tobemstomig, tobemstomig plus tiragolumab or atezolizumab plus tiragolumab versus nivolumab plus ipilimumab (control). We identify early signals of clinical activity for tobemstomig and translational correlates of pathological response in patients with clinical stage III melanoma.

## Results

### Study design, baseline clinicopathological characteristics and demographics

**Fig. 1:**
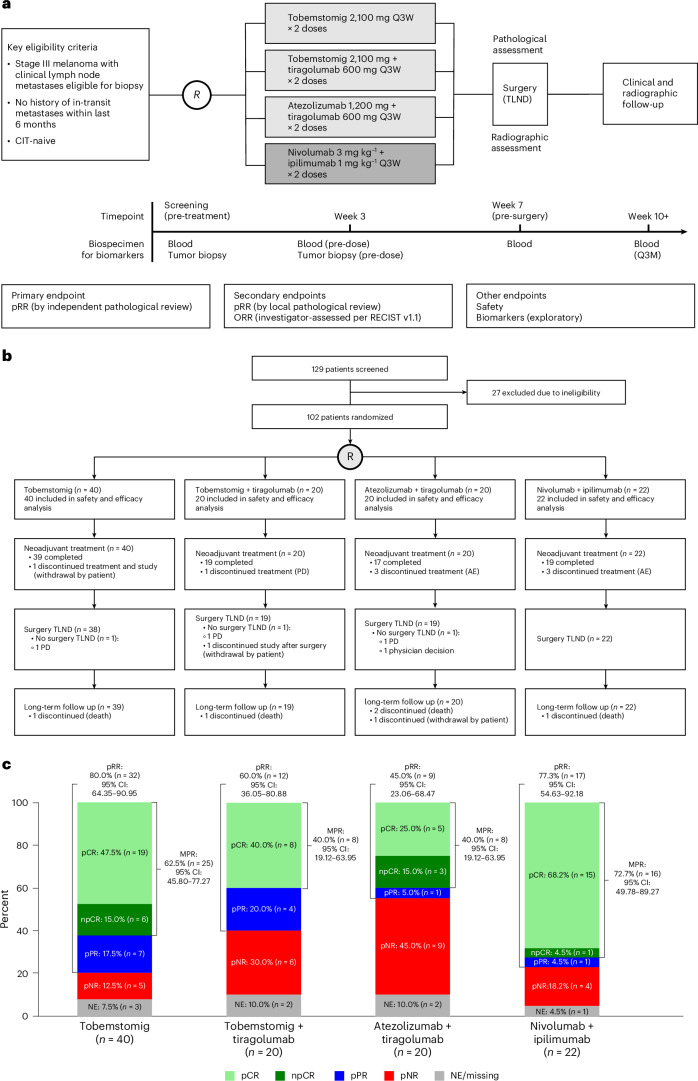
Morpheus-Melanoma study design, CONSORT diagram and pathological response. **a**, Eligible patients were randomized to receive one of the following neoadjuvant treatments Q3W for 6 weeks: tobemstomig 2,100 mg, tobemstomig 2,100 mg plus tiragolumab 600 mg, atezolizumab 1,200 mg plus tiragolumab 600 mg or control treatment of nivolumab 3 mg kg^−1^ plus ipilimumab 1 mg kg^−1^. Randomization was stratified by geographic region (Australia versus rest of the world) and baseline LDH (≤ULN versus >ULN). Therapeutic lymph node dissection occurred at week 7, and pathological response was evaluated according to INMC criteria. Tumor biopsies were obtained prior to treatment and at week 3 (pre-dose). Blood was collected for biomarker evaluation pre-treatment, at week 3, at the time of TLND (pre-TLND, week 7) and post-TLND at weeks 10 and 13. Dark gray denotes the control arm (nivolumab plus ipilimumab). **b**, Patient disposition by treatment arm, showing screening, randomization, completion of neoadjuvant therapy, TLND and long-term follow-up. **c**, Breakdown of pathological responses for the efficacy-evaluable population (*n* = 102). Pathological response by independent pathological review occurred in 32 patients (80.0%) in the tobemstomig arm, in 12 patients (60.0%) in the tobemstomig plus tiragolumab arm, in nine patients (45.0%) in the atezolizumab plus tiragolumab arm and in 17 patients (77.3%) in the nivolumab plus ipilimumab arm. pCR is defined as 0% viable tumor cells; npCR is defined as ≤10% viable tumor cells; pPR is defined as >10% to ≤50% viable tumor cells; and pNR is defined as >50% viable tumor cells. 95% CIs for rates were calculated using the Clopper–Pearson method. AE, adverse event; CI, confidence interval; CIT, cancer immunotherapy; NE, not evaluable; PD, progressive disease; Q3M, every 3 months; Q3W, every 3 weeks.

Morpheus-Melanoma was designed with the flexibility to open new treatment arms as new therapies became available, to close existing treatment arms that demonstrated minimal clinical activity or unacceptable toxicity or to expand enrollment in an experimental arm after promising initial signals. Patients 18 years of age or older with clinically detectable, RECIST-measurable, resectable stage III melanoma, with Eastern Cooperative Oncology Group (ECOG) performance status of 0 or 1 and no history of in-transit metastases within the last 6 months or prior radiotherapy or systemic cancer therapy for their disease, were randomized to receive preoperative tobemstomig, tobemstomig plus tiragolumab, atezolizumab plus tiragolumab or nivolumab plus ipilimumab (Fig. 1a).

The primary efficacy endpoint was pRR, defined as the percentage of pathological complete response (pCR), near pathological complete response (npCR) and pathological partial response (pPR), at the time of surgery, by independent pathological assessment. The secondary efficacy endpoints were pRR by local pathological assessment and investigator-assessed overall RECIST response rate (ORR). In addition, MPR (defined as pCR + npCR) was assessed. Safety endpoints included immune-related adverse events and delayed surgery. Exploratory biomarker analyses were performed.

Overall, 102 patients with stage III melanoma (excluding patients with mucosal and uveal melanoma or acral melanoma and/or in-transit metastases within 6 months prior to screening) were enrolled between 2 February 2022 and 8 August 2023. Forty patients were enrolled in the tobemstomig arm, which was expanded based on the totality of efficacy and safety data using prespecified criteria at interim analyses; 20 patients were enrolled in the tobemstomig plus tiragolumab arm; 20 patients were enrolled in the atezolizumab plus tiragolumab arm; and 22 patients were enrolled in the control arm of nivolumab plus ipilimumab, which was open throughout the study (Fig. 1b). Of the 102 enrolled patients, 94 (92.2%) completed the treatment period: 39 (97.5%) in the tobemstomig arm, 19 (95.0%) in the tobemstomig plus tiragolumab arm, 17 (85.0%) in the atezolizumab plus tiragolumab arm and 19 (86.4%) in the nivolumab plus ipilimumab arm. One patient in the tobemstomig arm withdrew from the study; one patient in the tobemstomig plus tiragolumab arm discontinued due to disease progression prior to surgery; and three patients each in the atezolizumab plus tiragolumab and nivolumab plus ipilimumab arms discontinued due to adverse events.

Most patients were White (76.5%) and male (66.7%), and patient age was 25–81 years across arms (Table 1). The median baseline target lesion sum of diameters was consistent across arms (Table 1).

**Table 1:** Characteristics of patients at baseline

	Tobemstomig (*n* = 40)	Tobemstomig + tiragolumab (*n* = 20)	Atezolizumab + tiragolumab (*n* = 20)	Nivolumab + ipilimumab (*n* = 22)
Age (years)				
Median (range)	66 (32–81)	58 (37–78)	59 (34–80)	50 (25–77)
Sex				
Male	31 (77.5%)	14 (70.0%)	12 (60.0%)	11 (50.0%)
Female	9 (22.5%)	6 (30.0%)	8 (40.0%)	11 (50.0%)
Race				
Asian	0	1 (5.0%)	0	0
Black or African American	0	1 (5.0%)	0	0
White	35 (87.5%)	14 (70.0%)	14 (70.0%)	15 (68.2%)
Unknown	5 (12.5%)	4 (20.0%)	6 (30.0%)	7 (31.8%)
Region^¶^				
Australia	18 (45.0%)	6 (30.0%)	7 (35.0%)	10 (45.5%)
Rest of the world	22 (55.0%)	14 (70.0%)	13 (65.0%)	12 (54.5%)
ECOG performance status				
0	35 (87.5%)	17 (85.0%)	18 (90.0%)	22 (100%)
1	5 (12.5%)	3 (15.0%)	2 (10.0%)	0
Primary diagnosis				
Local regional	34 (85.0%)	16 (80.0%)	15 (75.0%)	20 (90.9%)
Other (unknown)	6 (15.0%)	4 (20.0%)	5 (25.0%)	2 (9.1%)
Post-neoadjuvant treatment surgery - performed locations (TLND)^†^				
Axillary lymph node	13 (32.5%)	5 (25.0%)	9 (45.0%)	13 (59.1%)
Cervical/supraclavicular lymph node	13 (32.5%)	7 (35.0%)	6 (30.0%)	3 (13.6%)
Inguinal lymph node	11 (27.5%)	6 (30%)	4 (20.0%)	5 (22.7%)
Iliac lymph node	2 (5.0%)	3 (15.0%)	1 (5.0%)	0
Popliteal lymph node	0	0	0	1 (4.5%)
AJCC-8 stage at screening^‡^				
III	3 (7.5%)	2 (10.0%)	1 (5.0%)	1 (4.5%)
IIIA^#^	2 (5.0%)	0	0	2 (9.1%)
IIIB	15 (37.5%)	11 (55.0%)	10 (50.0%)	13 (59.1%)
IIIC	20 (50.0%)	6 (30.0%)	8 (40.0%)	5 (22.7%)
IIID	0	1 (5.0%)	1 (5.0%)	1 (4.5%)
LDH^¶^				
<1.5× ULN	38 (95.0%)	19 (95.0%)	20 (100%)	22 (100%)
1.5 to <2.5× ULN	2 (5.0%)	0	0	0
≥2.5× ULN	0	1 (5.0%)	0	0
Target lesions sum of diameters (mm)				
Median (range)	25.0 (15.0–154.0)	27.0 (15.0–75.0)	29.5 (15.0–76.0)	24.5 (15.0–55.0)
*BRAF* mutation status				
*n*	22	11	12	16
*V600E*	11 (50%)	6 (55%)	6 (50%)	7 (44%)
*V600K*	2 (9%)	1 (9%)	3 (25%)	1 (6%)
Other *BRAF* mutation	1 (5%)	1 (9%)	0	1 (6%)
Wild-type	8 (36%)	3 (27%)	3 (25%)	7 (44%)

^¶^Stratification factors.

^†^Four patients had surgery performed in two locations: one patient in the tobemstomig arm, two patients in the tobemstomig plus tiragolumab arm and one patient in the atezolizumab plus tiragolumab arm had surgery performed in the inguinal lymph node and iliac lymph node regions.

^‡^According to the 8th melanoma classification of the AJCC.

^#^Inclusion criteria have not been fulfilled.

At study closure (28 May 2024), median follow-up was 11.2 months in the tobemstomig arm, 15.2 months in the tobemstomig plus tiragolumab arm, 11.8 months in the atezolizumab plus tiragolumab arm and 13.6 months in the nivolumab plus ipilimumab arm.

### Efficacy

#### Pathological response

**Fig. 2:**
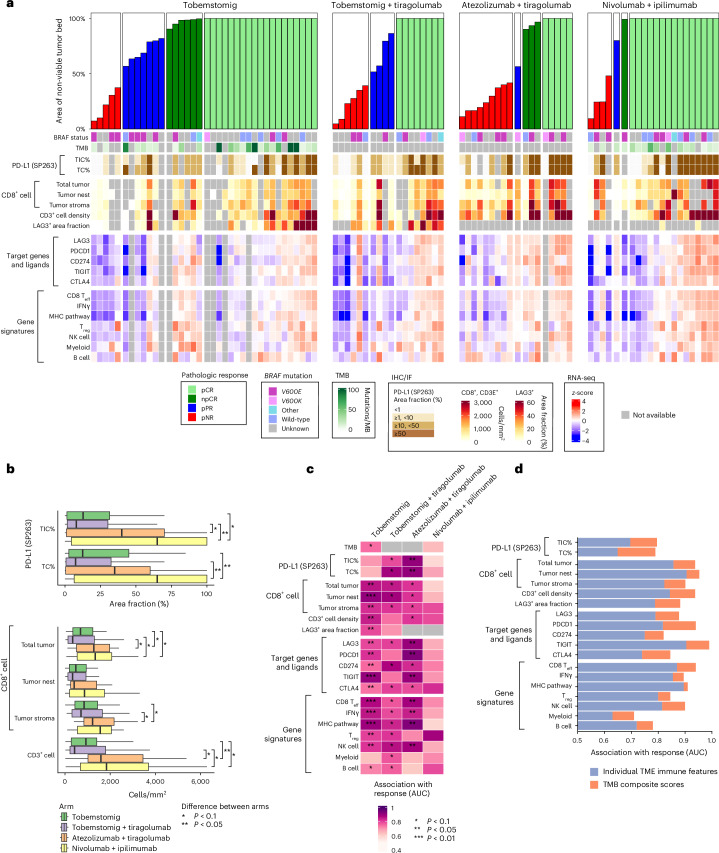
Baseline biomarker correlates of pathological response. **a**, Pathological response by independent pathological review, according to INMC guidelines, plotted as a waterfall showing the area of non-viable tumor bed at the time of surgery, alongside pre-treatment biomarkers, including *BRAF* mutation status, TMB, IHC, IF and RNA-seq. Patients with pCR are ordered according to tumor inflammation, as determined by the mean rank of all TME biomarkers shown. **b**, Pre-treatment prevalence of selected immune biomarkers in the TME of patients across treatment arms (*n* = 79 patients with IHC data). The box plots illustrate the distribution of biomarker levels, showing the median (central line), interquartile range (box) and data range (whiskers, up to 1.5 times the interquartile range). Asterisks denote statistically significant differences between arms (two-sided rank-sum test; see legend). **c**, Association between pre-treatment immune TME biomarkers and MPR (*n* = 87 patients with baseline data). The color gradient represents the area under the ROC curve (AUC), with values ranging from 0.5, indicating random prediction, to 1.0, indicating perfect prediction accuracy. Statistical significance is indicated using asterisks as defined in the legend (two-sided rank-sum test with Benjamini–Hochberg multiple testing correction). **d**, Improvement in predictive performance using composite scores of pre-treatment immune biomarkers combined with TMB. Stacked bar plots display the AUC for individual immune biomarkers (blue) and the enhanced AUC achieved by integrating these biomarkers with TMB into composite scores (red). IF, immunofluorescence; MB, megabase; NK; natural killer; ROC, receiver operating characteristic; TC, tumor cell; TIC, tumor-initiating cell.

**Extended Data Fig. 1:**
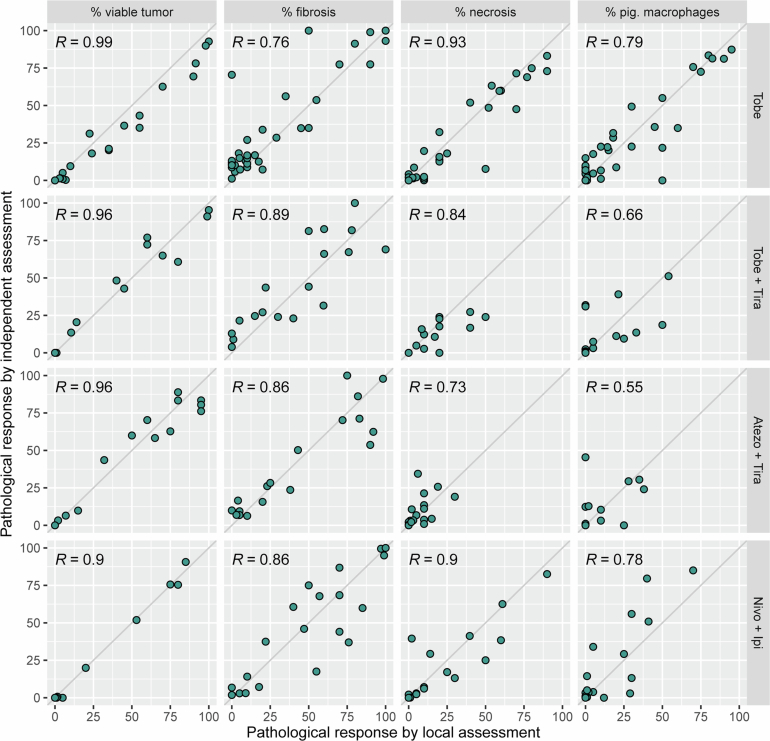
Correlation between independent and local pathological assessment. Scatterplots showing concordance between independent and local pathological assessments of therapeutic lymph node dissections from patients with Stage III melanoma treated with two cycles of tobemstomig (n = 36), tobemstomig plus tiragolumab (n = 18), atezolizumab plus tiragolumab (n = 18) and nivolumab plus ipilimumab (n = 21), respectively. Shown are percentages of viable tumor, fibrosis, necrosis and pigmented macrophages as a proportion of the tumor bed. Dots represent individual patients; diagonal lines indicate perfect agreement between the two assessments. R determined by Spearman’s rank correlation.

The efficacy-evaluable population comprised all 102 patients. Pathological response by independent pathological review occurred in 32 patients (80.0%) in the tobemstomig arm, in 12 patients (60.0%) in the tobemstomig plus tiragolumab arm, in nine patients (45.0%) in the atezolizumab plus tiragolumab arm and in 17 patients (77.3%) in the nivolumab plus ipilimumab arm (Figs. 1c and 2a and Supplementary Table 1). In the tobemstomig arm, 19 patients (47.5%) had pCR, six patients (15.0%) had npCR (MPR 62.5%) and seven patients (17.5%) had pPR. In the tobemstomig plus tiragolumab arm, eight patients (40.0%) had pCR (MPR 40.0%) and four patients (20.0%) had pPR. In the atezolizumab plus tiragolumab arm, five patients (25.0%) had pCR, three patients (15.0%) had npCR (MPR 40.0%) and one patient (5.0%) had pPR. In the nivolumab plus ipilimumab arm, 15 patients (68.2%) had pCR and one patient each (4.5% each) had npCR (MPR 72.7%) and pPR. Pathological response by independent and local assessment was consistent (Extended Data Fig. 1 and Supplementary Table 1).

#### ORR

**Extended Data Fig. 2:**
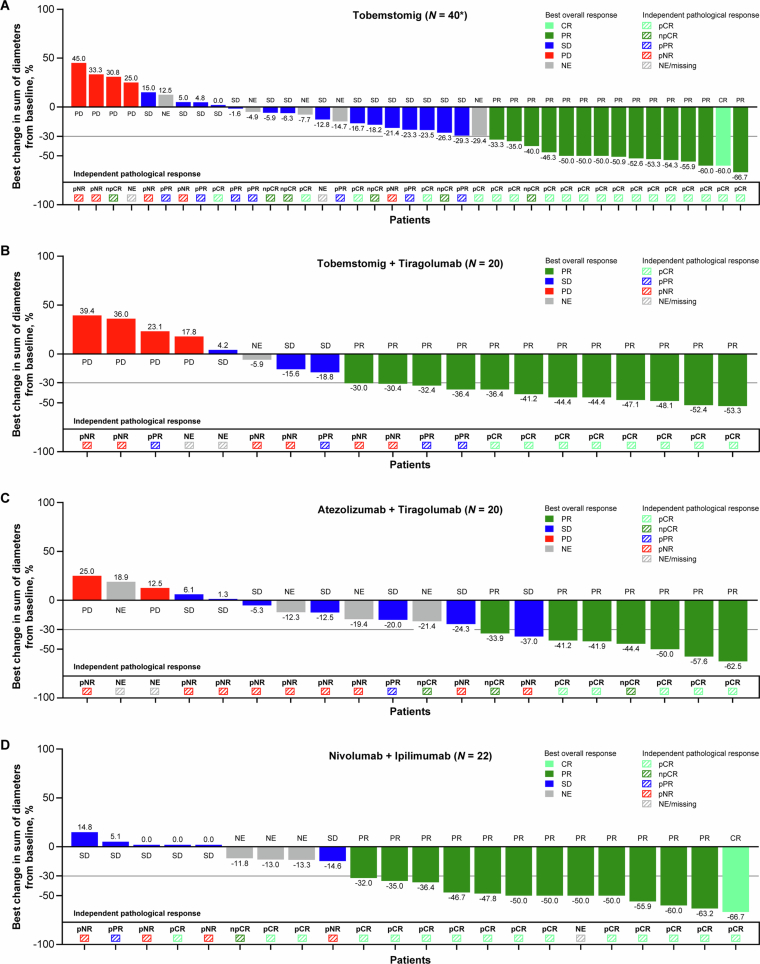
Best percentage change in tumor size (RECIST v1.1) and corresponding pathological response. Waterfall plots showing best percentage change in tumor size (sum of longest diameters) per patient in the (**a**) tobemstomig arm (n = 40*), (**b**) tobemstomig plus tiragolumab arm (n = 20), (**c**) atezolizumab plus tiragolumab arm (n = 20), and (**d**) nivolumab plus ipilimumab arm (n = 22) per RECIST v1.1 criteria. A percentage change above 0 indicates an increase in overall tumor size compared to baseline; a percentage change below 0 indicates a decrease in overall tumor size compared to baseline. The reference line at -30% indicates the RECIST response criteria for target lesions PR (partial response) as a decrease in the sum of diameters from baseline of at least 30%. *One patient had no post-baseline RECIST tumor assessment. CR, complete response; NE, not evaluable; npCR, near pathological complete response; pCR, pathological complete response; pNR, pathological no response; pPR, pathological partial response; PR, partial response; SD, stable disease.

The investigator-assessed ORR (per RECIST version 1.1) was 37.5% in the tobemstomig arm, 60.0% in the tobemstomig plus tiragolumab arm, 35.0% in the atezolizumab plus tiragolumab arm and 59.1% in the nivolumab plus ipilimumab arm (Extended Data Fig. 2). In general, ORR and pathological responses were concordant; however, radiographic assessment underestimated pathological response in some patients (Extended Data Fig. 2).

### Safety

The safety-evaluable population comprised all 102 patients. Overall, 36 patients (90.0%) in the tobemstomig arm, 18 patients (90.0%) in the tobemstomig plus tiragolumab arm, 19 patients (95.0%) in the atezolizumab plus tiragolumab arm and 19 patients (86.4%) in the nivolumab plus ipilimumab arm experienced at least one adverse event of any grade (Table 2). Time to onset and duration of each grade 3 or higher adverse event are provided in Supplementary Table 2.

**Table 2:** General safety summary (all events, with and without attribution to treatment)

	Tobemstomig (*n* = 40)	Tobemstomig + tiragolumab (*n* = 20)	Atezolizumab + tiragolumab (*n* = 20)	Nivolumab + ipilimumab (*n* = 22)
Total number of patients with at least one AE	36 (90.0%)	18 (90.0%)	19 (95.0%)	19 (86.4%)
Total number of AEs	178	103	61	133
Reasons for premature withdrawal from study drug				
AE	0	0	3 (15.0%)	3 (13.6%)
Progression of disease	0	1 (5.0%)	0	0
Withdrawal by patient	1 (2.5%)	0	0	0
Total number of patients with at least one				
Serious AE	9 (22.5%)	6 (30.0%)	3 (15.0%)	4 (18.2%)
Grade 3–5 AE	8 (20.0%)	6 (30.0%)	1 (5.0%)	6 (27.3%)
AE with fatal outcome (worst grade: 5)	0	0	0	0
Worst grade: 4	3 (7.5%)	0	0	2 (9.1%)
Worst grade: 3	5 (12.5%)	6 (30.0%)	1 (5.0%)	4 (18.2%)
AE requiring systemic corticosteroid and/or other immunosuppressive treatment	11 (27.5%)	6 (30.0%)	6 (30.0%)	9 (40.9%)
TRAE	34 (85.0%)	17 (85.0%)	15 (75.0%)	18 (81.8%)
Treatment-related serious AE	3 (7.5%)	3 (15.0%)	2 (10.0%)	3 (13.6%)
Treatment-related grade 3–5 AE	1 (2.5%)	3 (15.0%)	0	5 (22.7%)
TRAE leading to withdrawal from any treatment	0	0	3 (15.0%)	3 (13.6%)
TRAE leading to dose modification/interruption	4 (10.0%)	3 (15.0%)	0	1 (4.5%)
Immune-mediated AEs by medical concept				
Total number of patients with at least one immune-mediated AE	29 (72.5%)	13 (65.0%)	8 (40.0%)	15 (68.2%)
Most common immune-mediated AEs (≥10% in any arm)				
Immune-mediated rash	12 (30.0%)	6 (30.0%)	5 (25.0%)	7 (31.8%)
Immune-mediated hepatitis	7 (17.5%)	3 (15.0%)	1 (5.0%)	8 (36.4%)
Immune-mediated hyperthyroidism	7 (17.5%)	6 (30.0%)	3 (15.0%)	4 (18.2%)
Immune-mediated hypothyroidism	6 (15.0%)	1 (5.0%)	1 (5.0%)	2 (9.1%)
Infusion-related reactions	6 (15.0%)	2 (10.0%)	0	2 (9.1%)
Immune-mediated pancreatitis	5 (12.5%)	1 (5.0%)	0	2 (9.1%)
TLND status				
Completed	38 (95.0%)	19 (95.0%)	18 (90.0%)	22 (100%)
Not performed	2 (5.0%)	1 (5.0%)	2 (10.0%)	0
Reason why TLND was not performed				
*n*	2	1	2	0
Physician decision	0	0	1 (5.0%)	0
Progressive disease	1 (2.5%)	1 (5.0%)	1 (5.0%)	0
Withdrawal by patient	1 (2.5%)	0	0	0
TLND delay category				
Within 2 weeks as scheduled	35 (87.5%)	18 (90.0%)	18 (90.0%)	18 (81.8%)
More than 2 weeks delayed	3 (7.5%)	1 (5.0%)	0	4 (18.2%)
Reason for more than 2 weeksʼ TLND delay				
*n*	3	1	0	4
AE	2 (5.0%)	1 (5.0%)	0	3 (13.6%)
Other	1 (2.5%)	0	0	1 (4.5%)
TRAEs leading to TLND delay				
*n*	1	1	0	3
Hyperthyroidism	1 (2.5%)	1 (5.0%)	0	0
Meningitis aseptic	0	0	0	1 (4.5%)
Pneumonitis	0	0	0	1 (4.5%)
Tachycardia	0	0	0	1 (4.5%)
Non-related AEs leading to TLND delay				
*n*	1	0	0	0
SARS-CoV-2 test positive	1 (2.5%)	0	0	0

AE, adverse event.

TRAEs were experienced by 34 patients (85.0%) treated with tobemstomig, by 17 patients (85.0%) treated with tobemstomig plus tiragolumab, by 15 patients (75.0%) treated with atezolizumab plus tiragolumab and by 18 patients (81.8%) treated with nivolumab plus ipilimumab. The most common TRAEs (≥20% in any arm) were fatigue (30.0% versus 25.0% versus 15.0% versus 31.8%), hyperthyroidism (17.5% versus 30.0% versus 15.0% versus 18.2%), rash (17.5% versus 10.0% versus 5.0% versus 27.3%), pruritus (15.0% versus 15.0% versus 5.0% versus 36.4%) and asthenia (5.0% versus 5.0% versus 20.0% versus 9.1%) in the tobemstomig, tobemstomig plus tiragolumab, atezolizumab plus tiragolumab and nivolumab plus ipilimumab arms, respectively.

One patient (2.5%) in the tobemstomig arm, three patients (15.0%) in the tobemstomig plus tiragolumab arm, no patients in the atezolizumab plus tiragolumab arm and five patients (22.7%) in the nivolumab plus ipilimumab arm experienced a grade 3 or higher TRAE. There were no treatment-related deaths in any of the treatment arms.

Protocol-predefined immune-mediated adverse events are described in Table 2 and Supplementary Table 3.

#### Therapeutic lymph node dissection rates

There were 38 patients (95.0%) treated with tobemstomig, 19 patients (95.0%) treated with tobemstomig plus tiragolumab, 18 patients (90.0%) treated with atezolizumab plus tiragolumab and 22 patients (100%) treated with nivolumab plus ipilimumab who underwent a therapeutic lymph node dissection (TLND). TLND was performed within 2 weeks of the second cycle of neoadjuvant therapy, as scheduled, for most patients in each arm but was delayed by more than 2 weeks in eight patients (Table 2). TLND was not performed in five patients due to progressive disease (one patient each in the tobemstomig, tobemstomig plus tiragolumab and atezolizumab plus tiragolumab arms), patient withdrawal (one patient in the tobemstomig arm) and physician decision (one patient in the atezolizumab plus tiragolumab arm). Of the eight patients who had TLND delayed by more than 2 weeks, most delays were due to adverse events. Adverse events leading to TLND delay were hyperthyroidism and a SARS-CoV-2-positive test in the tobemstomig arm (one patient each), hyperthyroidism in the tobemstomig plus tiragolumab arm (one patient) and aseptic meningitis, pneumonitis and tachycardia in the nivolumab plus ipilimumab arm (one patient each). No patients in the atezolizumab plus tiragolumab arm had TLND delayed by more than 2 weeks.

### Biomarkers

#### Baseline tumor microenvironment biomarker distributions by treatment arm

**Extended Data Fig. 3:**
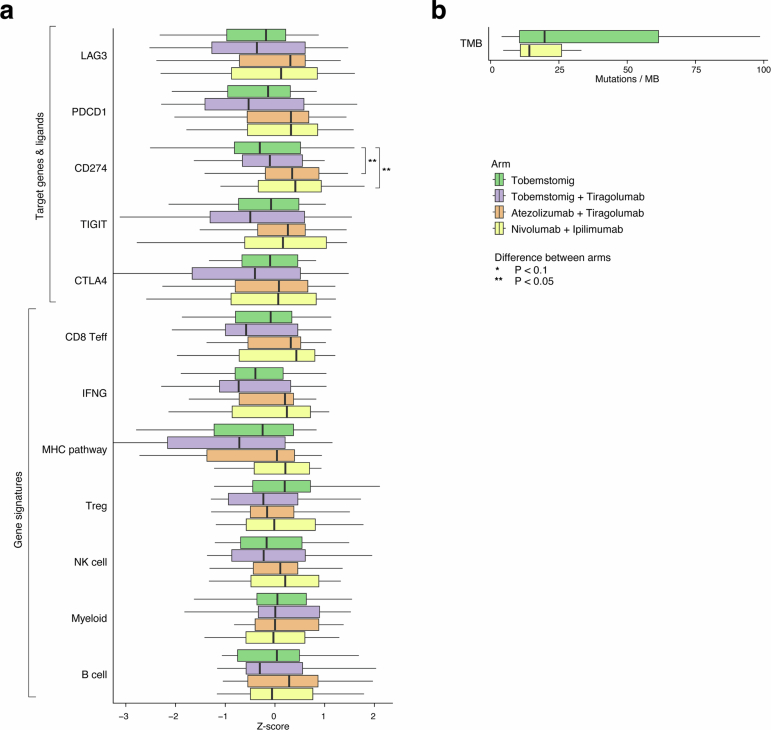
Baseline prevalence of gene and gene signature expression and TMB in tumor tissue. Prevalence of pre-treatment (**a**) immune gene and gene signatures (n = 79) and (**b**) tumor mutational burden (TMB, n = 41) in the tumor microenvironment in patients according to treatment arm. Boxplots illustrate the distribution of biomarker levels, showing the median (central line), interquartile range (box), and data range (whiskers). Asterisks denote statistically significant differences between arms as defined in the legend (two-sided rank-sum test). IFNG, interferon gamma; LAG-3, lymphocyte-activation gene 3; MHC, major histocompatibility; NK; natural killer; TMB, tumor mutational burden; Treg, regulatory T cell.

Overall, 90 of 102 patients (88.2%) had pre-treatment biopsies with sufficient tumor content for baseline biomarker evaluation. At study entry, canonical markers of an inflamed tumor microenvironment (TME), such as PD-L1 protein and gene expression and CD3^+^ and CD8^+^ T cell density in tumor stroma and tumor nests, were lower in patients in the tobemstomig arm versus the nivolumab plus ipilimumab arm (Fig. 2a,b and Extended Data Fig. 3). Tumor mutational burden (TMB; evaluated only in patients treated with tobemstomig and nivolumab plus ipilimumab based on favorable efficacy results) and the prevalence of *BRAF V600E* and *V600K* mutations were similar in patients in the tobemstomig arm and the nivolumab plus ipilimumab arm. The level of baseline tumor T cell infiltration was similar for patients enrolled in the tobemstomig and tobemstomig plus tiragolumab arms.

#### Association of baseline TME biomarkers and pathological response

**Extended Data Fig. 4:**
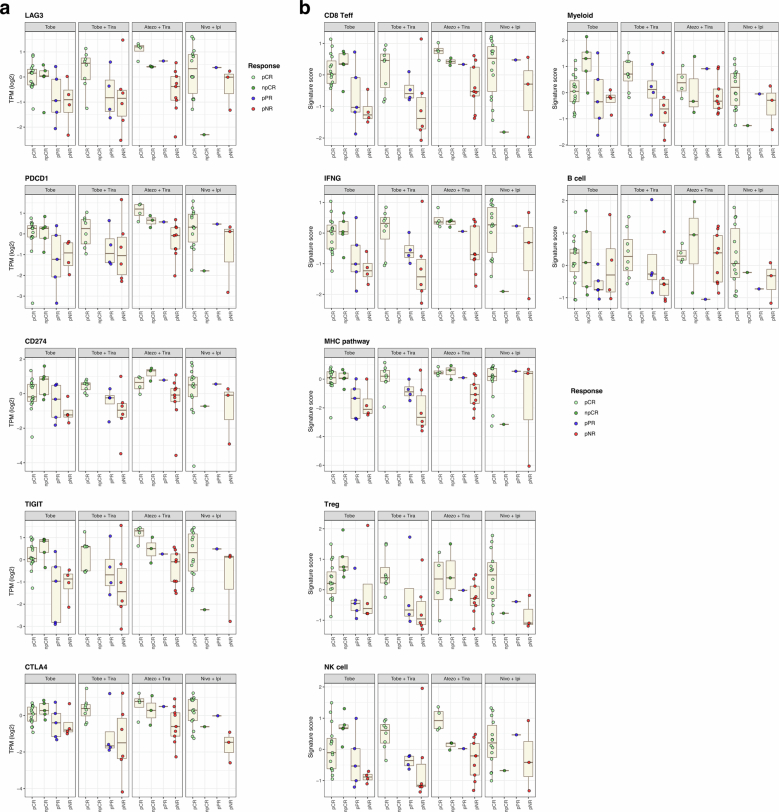
Baseline tumor RNAseq biomarkers by treatment. Boxplots showing expression of RNAseq-based biomarkers in pre-treatment tumor samples (n = 79), including (**a**) individual immune-related genes and (**b**) immune-related gene signatures in the tumor microenvironment. Patients are grouped by treatment and colored by pathological response. Boxplots illustrate the median (central line), interquartile range (box), minima and maxima (whiskers, up to 1.5 times the interquartile range), with data points beyond this limit shown as individual outliers. Individual patients (dots) colored by pathological response. IFNG, interferon gamma; LAG-3, lymphocyte-activation gene 3; MHC, major histocompatibility; NK; natural killer; npCR, near pathological complete response; pCR, pathological complete response; pNR, pathological no response; pPR, pathological partial response; TMB, tumor mutational burden; Treg, regulatory T cell.

**Extended Data Fig. 5:**
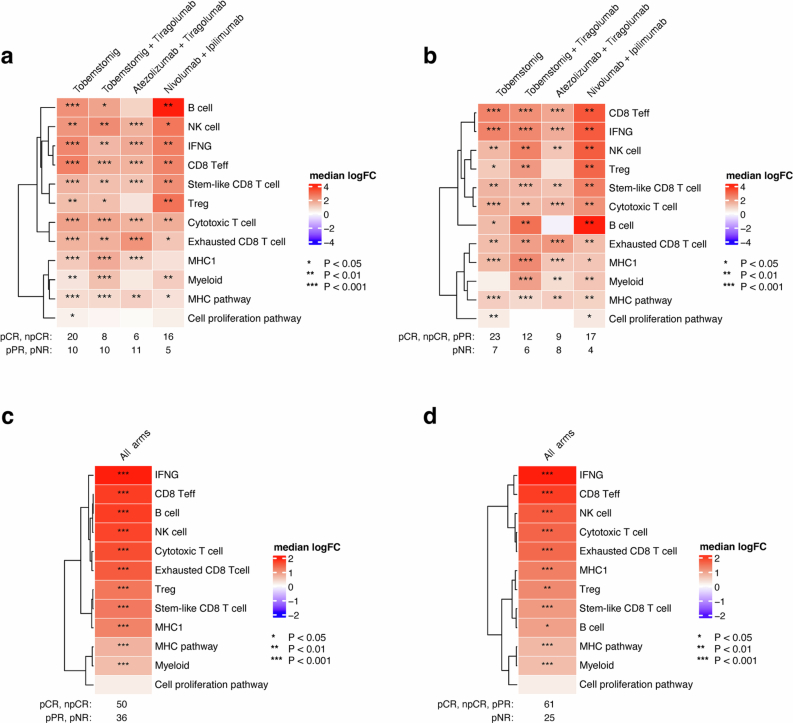
Baseline differential gene expression analysis of pathological responders versus non-responders. Differential gene expression heatmap showing baseline immune genes and gene signatures and their relative association with pathological response stratified by treatment (**a**, **b**) or pooled across treatments (**c**, **d**). Association is evaluated either by comparing major pathological responders versus others (**a**, **c**) or pathological responders versus non-responders (**b**, **d**). IFNG, interferon gamma; LAG-3, lymphocyte-activation gene 3; MHC, major histocompatibility; NK; natural killer; TMB, tumor mutational burden; Treg, regulatory T cell.

Several baseline immune features in the TME were associated with pathological response to treatment. In the tobemstomig arm, baseline tumor-infiltrating CD8^+^ T cell density (in tumor nests and stroma), CD3^+^ T cell density, LAG-3 protein expression, immune-related genes and gene signatures (including *LAG-3*, *PDCD1*, CD274, CD8 T_eff_, IFNγ pathway and major histocompatibility complex (MHC) pathway) were associated with MPR (*P* < 0.05) (Fig. 2c, Extended Data Fig. 4 and Supplementary Fig. 1). Similar associations were observed with the other treatments (Fig. 2c, Extended Data Fig. 4 and Supplementary Fig. 1) and were consistent irrespective of the response threshold (MPR or any pathological response; Extended Data Fig. 5 and Supplementary Fig. 2).

*BRAF V600E* mutation status was not associated with pathological response to any treatments, although there was a trend for a greater proportion of non-responders in patients with *BRAF V600E* versus others in the tobemstomig arm (Fig. 2a). Among the seven patients with *BRAF V600K* mutations, five were pathological responders, one was a pathological non-responder and one had no pathological response evaluation. Patients with *BRAF V600K* mutations had similar levels of baseline tumor CD8 T cell infiltration to patients with *BRAF V600E* mutations.

**Extended Data Fig. 6:**
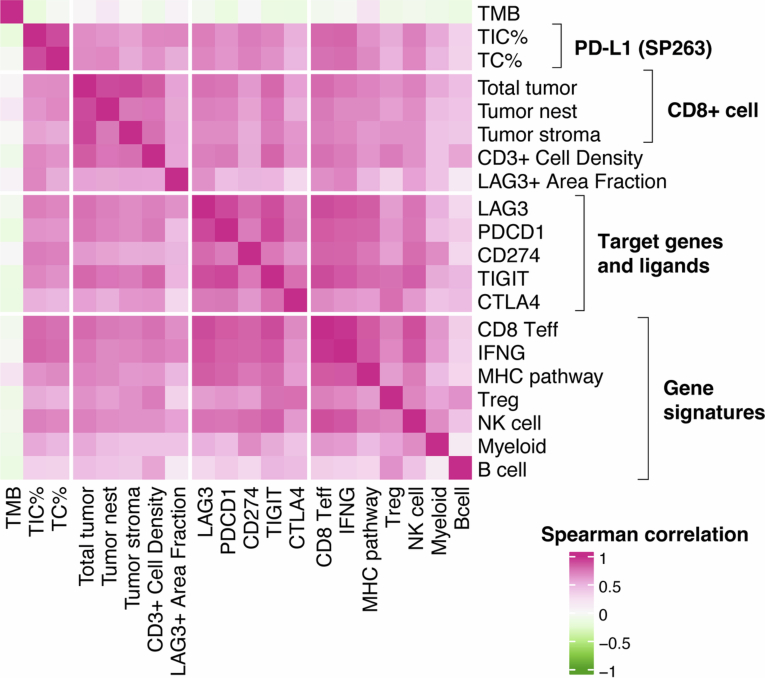
Baseline tumor biomarker correlation matrix. Correlation of RNA-seq, immunohistochemistry-, immunofluorescence- and whole exome sequencing-based biomarkers in pre-treatment lymph node biopsies from patients treated with tobemstomig, tobemstomig plus tiragolumab, atezolizumab plus tiragolumab and nivolumab plus ipilimumab (n = 79). Tumor mutational burden (TMB, n = 41) was evaluated only in patients treated with tobemstomig or nivolumab plus ipilimumab. IFNG, interferon gamma; LAG-3, lymphocyte-activation gene 3; MHC, major histocompatibility; NK; natural killer; PD-L1, programmed death-ligand 1; RNA-seq; RNA sequencing; TC, tumor cells; TIC, tumor-initiating cells; TMB, tumor mutational burden; Treg, regulatory T cell.

**Extended Data Fig. 7:**
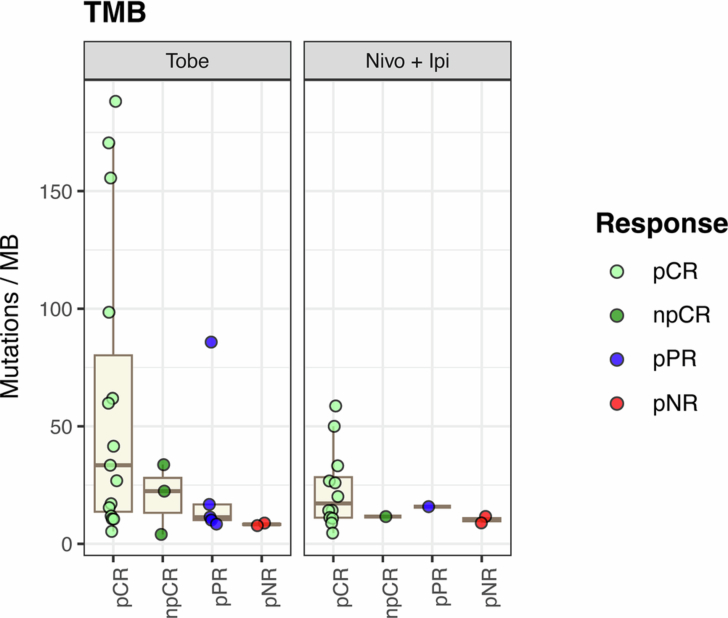
Baseline tumor mutational burden by treatment. Boxplots showing distribution of tumor mutational burden (TMB) in pre-treatment tumor tissue of patients treated with tobemstomig (n = 25) or nivolumab plus ipilimumab (n = 16). Boxplots illustrate the median (central line), interquartile range (box), minima and maxima (whiskers, up to 1.5 times the interquartile range), with data points beyond this limit shown as individual outliers. Individual patients (dots) colored by pathological response. npCR, near pathological complete response; pCR, pathological complete response; pNR, pathological no response; pPR, pathological partial response.

**Extended Data Fig. 8:**
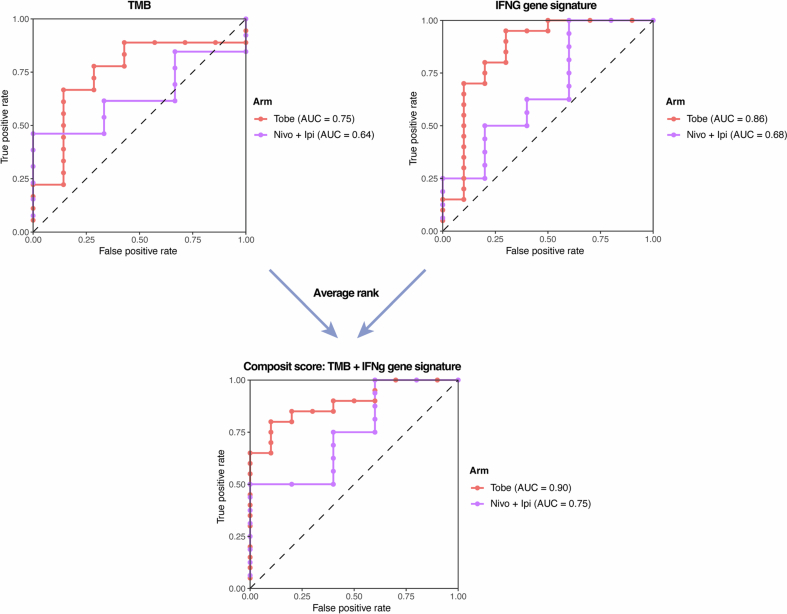
Baseline ROC curves for predictive performance of IFN-γ and TMB scores (individual and composite scores for major pathological response [MPR]). Receiver operating characteristic (ROC) curves evaluating the ability of pre-treatment TMB (left panel), IFN-γ gene signature score (right panel), and a rank-based TMB + IFN-γ composite score (bottom panel) to predict MPR. Each panel displays ROC curves for two treatment arms: tobemstomig monotherapy (red) and nivolumab plus ipilimumab combination therapy (purple), with corresponding area under the curve (AUC) values annotated. Composite scores were derived from normalized percentile ranks of IFN-γ (n = 79) and TMB (n = 41) (see Methods). Curves demonstrate enhanced predictive power when integrating immune biomarkers with TMB. AUC, area under the receiver operating characteristic curve; IFNg, interferon gamma; TMB, tumor mutational burden.

Immune-related genes and proteins indicative of TME inflammation were correlated at baseline and were independent of TMB (Extended Data Fig. 6). TMB was associated with pathological response to tobemstomig, albeit to a lesser degree than other inflammatory TME immune biomarkers evaluated (Fig. 2c and Extended Data Fig. 7). The predictive potential of individual biomarkers improved when combined with TMB (Fig. 2d and Extended Data Fig. 8).

#### Immune cell dynamics in the TME

**Fig. 3:**
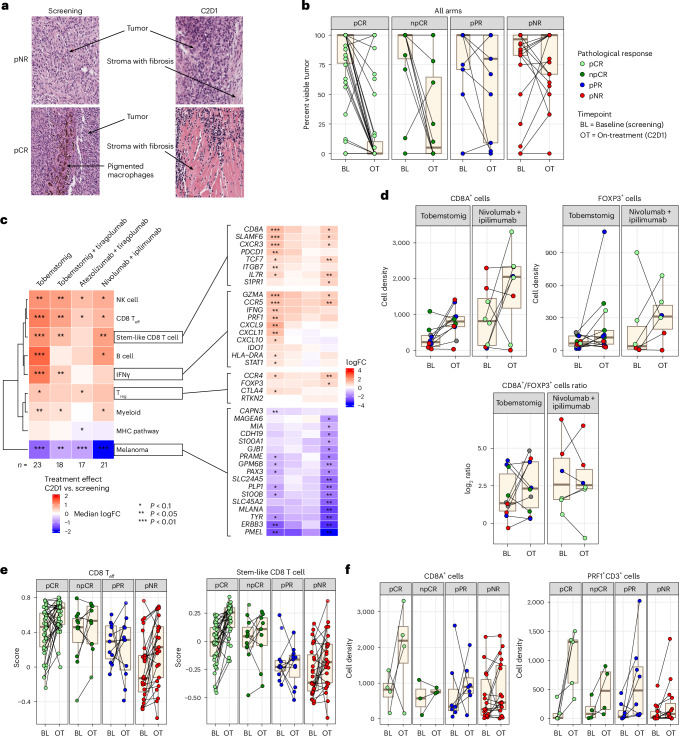
Immune dynamics in the TME. Immune dynamics in the TME, relevant to the drug MOA, were evaluated in pre-treatment and on-treatment lymph node tumor tissue from patients with clinical stage III melanoma treatment with neoadjuvant immunotherapy. **a**, Representative histologic H&E stains of metastatic pre-treatment and on-treatment tumor tissue from pNR and pCR patients after one cycle of tobemstomig treatment, demonstrating reduction in viable tumor and increases in fibrosis in a patient with pCR compared to a patient with pNR. **b**, Box plot showing the percentage of viable tumor in pre-treatment and on-treatment tumor tissue from patients in each pathological response category after one treatment cycle (*n* = 79). Patients from different treatment arms are pooled. **c**, Heatmap representation of changes in immune and tumor genes and gene signatures in the TME on-treatment compared to pre-treatment (left) with zoom out on the individual genes comprising stem-like T cells, IFNγ, T_reg_ cells and melanoma gene signatures, respectively (right). Patients are grouped according to treatment. Statistical significance is indicated using asterisks as defined in the legend (two-sided rank-sum test with Benjamini–Hochberg multiple testing correction). **d**, Box plot representation of IHC/IF-derived CD8^+^ T cell and FOXP3 density and CD8/FOXP3 ratio in pre-treatment and on-treatment metastatic tumor biopsies from patients treated with tobemstomig (*n* = 13) or nivolumab plus ipilimumab (*n* = 8). **e**,**f**, Pooled analysis across all study treatments of CD8 T cell dynamics on-treatment according to pathological response. CD8 T_eff_ and stem-like CD8 T cell signatures were derived from tumor bulk RNA-seq (*n* = 79) (**e**), whereas CD8^+^ and CD3^+^Perforin^+^ T cell densities were assessed by IHC and IF, respectively (*n* = 32) (**f**). Box plots illustrate the median (central line), interquartile range (box) and minima and maxima (whiskers, up to 1.5 times the interquartile range), with data points beyond this limit shown as individual outliers. Individual patients (dots) are colored by pathological response. FC, fold change; PRF1, Perforin; IF, immunofluorescence; NK; natural killer; TC, tumor cell.

Paired metastatic lymph node biopsies were obtained from 79 patients (77.5%) before and after one treatment cycle and were evaluated for pharmacodynamic biomarkers relevant to drug mode of action (MOA). In many patients, little to no viable tumor was observed in on-treatment biopsies obtained after a single treatment cycle at cycle 2, day 1 (C2D1). These patients demonstrated distinct histologic features, including extensive levels of fibrosis and immune infiltration, compared to others; ultimately, these patients were identified as MPR at the time of TLND (Fig. 3a,b).

Treatment led to the upregulation of immune-related gene signatures in the TME (IFNγ pathway, CD8 T_eff_, MHC pathway) and downregulation of melanoma-associated gene signatures after one treatment cycle. The most notable transcriptional changes in the TME on-treatment were observed with tobemstomig and nivolumab plus ipilimumab (Fig. 3c and Supplementary Fig. 3).

To evaluate the proposed MOA of tobemstomig to reinvigorate TILs and bypass T_reg_ cells in the TME, relevant immune-related gene signatures were evaluated and compared to those elicited in response to nivolumab plus ipilimumab. CD8 T_eff_ cells, stem-like T cells and IFNγ pathway gene signatures increased with tobemstomig and nivolumab plus ipilimumab treatment (*P* < 0.01) (Fig. 3c). T_reg_ gene signatures increased with tobemstomig and nivolumab plus ipilimumab treatment (Fig. 3c and Supplementary Fig. 3). Consistent with gene expression data, CD8 TIL density (including in tumor nests and stroma) and proliferating (CD8^+^Ki67^+^) and cytotoxic (CD3^+^Perforin^+^) T cells increased with tobemstomig treatment (*P* < 0.05) (Fig. 3d and Supplementary Fig. 4). The ratio of CD8 to FOXP3 tended to increase with tobemstomig (not significant) but did not change with nivolumab plus ipilimumab treatment (Fig. 3d).

No significant differences were observed in immune cell dynamics after tobemstomig versus tobemstomig plus tiragolumab treatment, based on tumor whole transcriptome.

**Extended Data Fig. 9:**
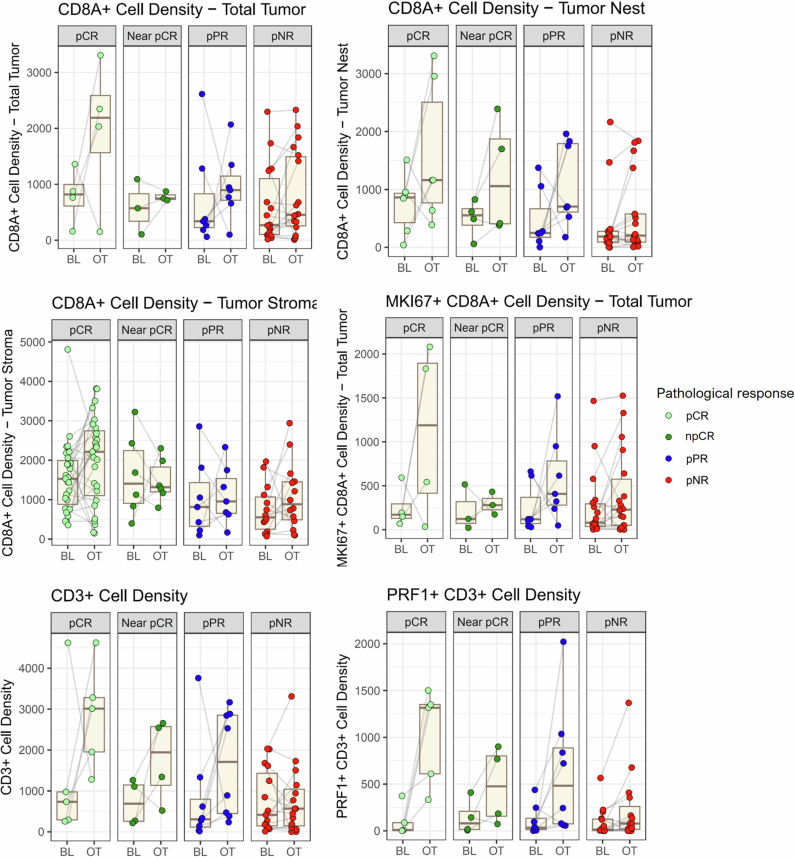
Tumor-infiltrating T cell dynamics by pathological response. Pooled analysis of immune dynamics pre-treatment (screening) and after one treatment cycle (C2D1) across treatment groups (n = 32). Boxplots show immunohistochemistry and immunofluorescence -derived densities of tumor-infiltrating T cell subsets, including CD8+ (viable tumor, tumor nest and tumor stroma), CD8+ Ki67+, CD3+, CD3+ Perforin+ cells. Boxplots illustrate the median (central line), interquartile range (box), minima and maxima (whiskers, up to 1.5 times the interquartile range), with data points beyond this limit shown as individual outliers. Individual patients (dots) colored by pathological response. BL, baseline; C2D1, cycle 2 day 1; npCR, near pathological complete response; OT, one treatment; pCR, pathological complete response; pNR, pathological no response; pPR, pathological partial response.

Because all CPIs evaluated in this study aim to reinvigorate TILs, and to overcome the limited sample size within individual treatment arms, we pooled patients across treatments and evaluated the association between tumor-infiltrating T cell dynamics with pathological response. In general, responders had higher on-treatment immune gene and protein expression versus non-responders. Interestingly, some patients with low or medium levels of baseline CD8 T cell density and gene expression showed robust increases on-treatment, in absence of clinical benefit (that is, were pathological non-responders (pNR)), potentially reflecting underlying T cell intrinsic or extrinsic resistance mechanisms that may hinder generation of an effective antitumor immune response (Fig. 3e,f and Extended Data Fig. 9).

#### Circulating tumor DNA dynamics

Circulating tumor DNA (ctDNA) was evaluated over time in patients treated with tobemstomig and nivolumab plus ipilimumab (*n* = 39), based on the favorable efficacy observed for these treatments compared to others. Pre-treatment ctDNA was detected in 35 of 39 patients (89.7%), despite early disease stage.

**Fig. 4:**
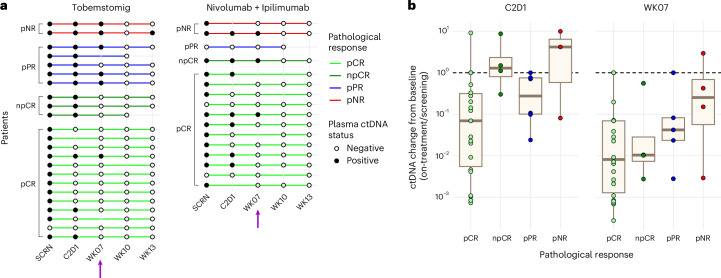
ctDNA dynamics. Longitudinal ctDNA dynamics assessed over time in patients treated with tobemstomig or nivolumab plus ipilimumab. **a**, Swimmer plot depicting ctDNA status over patient visit from screening (pre-treatment), C2D1, week 7 (pre-surgery) and weeks 10 and 13 (post-surgery) (*n* = 39). Patients are grouped by treatment and ordered by pathological response within each treatment group. Purple arrow indicates the time of TLND (week 7). **b**, Box plot representing change in ctDNA levels at the pre-surgery timepoints, C2D1 and week 7, compared to pre-treatment levels for pathological response categories (*n* = 39). Ratio represents the mean tumor molecules per milliliter of plasma at on-treatment compared to baseline. Patients from different treatment arms are pooled. Box plots illustrate the median (central line), interquartile range (box) and minima and maxima (whiskers, up to 1.5 times the interquartile range), with data points beyond this limit shown as individual outliers. Individual patients (dots) are colored by pathological response. SCRN, screening; WK, week.

**Extended Data Fig. 10:**
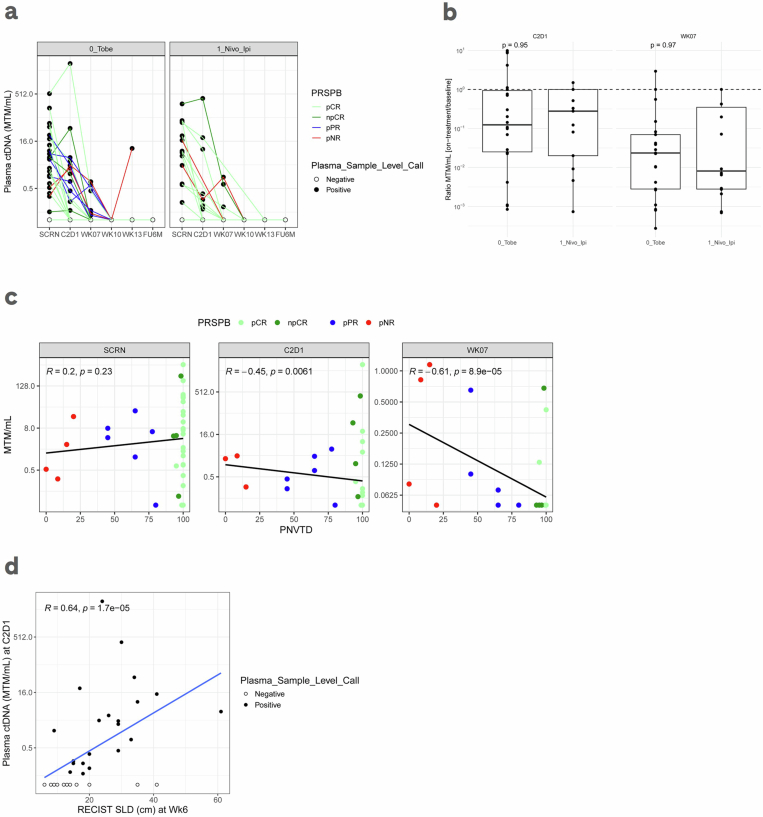
ctDNA dynamics and association with pathological response. (**a**) Longitudinal ctDNA levels across timepoints (screening (pre-treatment), cycle 2 day 1 (C2D1), Week 7 (pre-TLND) and Weeks 10 and 13 (post-TLND)) in patients treated with tobemstomig or nivolumab plus ipilimumab (n = 39). (**b**) Box plot showing ctDNA changes at C2D1 and Week 7, relative to pre-treatment, stratified by treatment (n = 39). Ratios represent the mean tumor molecules per mL (MTM/ml) plasma on-treatment vs. pre-treatment. Patients are pooled across pathological response categories; dots indicate individual patients colored by pathological response. P-values were calculated using the two-sided Wilcoxon rank-sum Test (no correction for multiple testing). Boxplots illustrate the median (central line), interquartile range (box), minima and maxima (whiskers, up to 1.5 times the interquartile range), with data points beyond this limit shown as individual outliers. (**c**) Correlation of ctDNA with vs. percentage of non-viable tumor deposit (PNVTD) pre-treatment, C2D1 and Week 7. (**d**) Correlation between ctDNA at C2D1 RECIST sum of longest diameter (SLD). R and p-value determined using Spearman’s rank correlation. C2D1, cycle 2 day 1; ctDNA, circulating tumor DNA; npCR, near pathological complete response; pCR, pathological complete response; pNR, pathological no response; pPR, pathological partial response; TLND, therapeutic lymph node dissection.

Among patients with detectable ctDNA at baseline, 68% of those with a pathological response (21/31: pCR 17/22; npCR 3/4; pPR 1/5) achieved ctDNA clearance by week 7 pre-surgery versus one of four patients (25%) with pNR (Fig. 4a and Extended Data Fig. 10a). Furthermore, ctDNA clearance was observed in 50.0% (11/22) of patients with pCR after one cycle of CPI treatment and increased to 77.3% (17/22) after two cycles of CPI treatment.

In patients with pCR and npCR, with no ctDNA clearance prior to surgery, significant decreases in ctDNA were nevertheless observed in all cases at week 7 (100%) versus pre-treatment levels. Patients with pCR had lower on-treatment ctDNA levels relative to baseline compared to other patients (C2D1: *P* = 0.01; week 7: *P* = 0.09) (Fig. 4b).

Pre-surgery ctDNA longitudinal dynamics were similar in patients treated with tobemstomig and nivolumab plus ipilimumab (C2D1: *P* = 0.95; week 7: *P* = 0.97) (Extended Data Fig. 10b) and correlated with the percentage of non-viable tumor deposits at the time of surgery (C2D1: *r* = −0.45, *P* = 0.0061; week 7: *r* = −0.61, *P* < 0.0005) (Extended Data Fig. 10c) and with radiographic response by RECIST at week 6 (C2D1: *r* = 0.64, *P* < 0.005) (Extended Data Fig. 10d).

Most patients with detectable ctDNA at baseline were ctDNA negative after surgery, both at week 10 and at week 13, with no disease recurrence. The only exception was one patient with pNR with an increase in ctDNA levels at week 13 and concomitant disease recurrence (Fig. 4a and Extended Data Fig. 10b). Among patients with no detectable ctDNA at baseline (4/39: three pCR, one pPR), all remained ctDNA negative throughout the subsequent visits evaluated.

## Discussion

Neoadjuvant tobemstomig demonstrated encouraging clinical efficacy in patients with resectable clinical stage III melanoma with a similar pRR to patients treated with nivolumab plus ipilimumab (80.0% versus 77.3%), despite a more immune-rich pre-treatment TME in patients receiving nivolumab plus ipilimumab. MPR was 62.5% after tobemstomig treatment and 72.7% after nivolumab plus ipilimumab treatment. The trial was not designed to formally test the statistical difference in efficacy between treatment arms. Instead, Bayesian posterior probability of equal or better pRR between experimental and control arms, together with a benefit–risk assessment, guided the decision to expand enrollment in the tobemstomig arm.

Although tobemstomig is administered as a single agent, it targets dual immune checkpoint blockade, effectively mimicking the benefits of a combination therapy. Compared to the combination of nivolumab plus ipilimumab, fewer patients treated with tobemstomig experienced grade 3 or higher TRAEs (22.7% versus 2.5%). The addition of tiragolumab to tobemstomig or to atezolizumab showed a lower pRR versus the control treatment and resulted in higher toxicity versus tobemstomig alone; the increased toxicity is not unexpected given that the former are effectively triple CPI combinations.

The phase 3 NADINA trial demonstrated improved efficacy but increased toxicity for neoadjuvant nivolumab plus ipilimumab versus adjuvant nivolumab, with 29.7% of patients treated with nivolumab plus ipilimumab experiencing grade 3 or higher TRAEs^5^. The results from our study are broadly consistent with the NADINA trial for efficacy (pRR 77.3% versus 67% and MPR 72.7% versus 59.0%, respectively) and safety^5^. The higher efficacy observed in our study versus NADINA may be attributed to the high baseline infiltration status of patients enrolled in the nivolumab plus ipilimumab treatment arm in our study, although no direct comparison across studies is feasible. In addition, the earlier phase of our study and the fact that it was executed across fewer sites could also account for differences in efficacy versus the larger, later-stage NADINA trial.

Several alternative neoadjuvant regimens have been investigated for increased efficacy and reduced toxicity versus nivolumab plus ipilimumab. In a phase 2 trial of patients with resectable clinical stage III or oligometastatic stage IV melanoma, neoadjuvant relatlimab, an anti-LAG-3 monoclonal antibody, combined with nivolumab and adjuvant combination therapy demonstrated a pRR of 70.0% and an MPR of 63%, with no grade 3 or higher immune-related adverse events during neoadjuvant treatment^10^. In the phase 1/2 KEYMAKER-U02 substudy 02C of neoadjuvant treatment followed by adjuvant pembrolizumab in patients with stage IIIB–D melanoma, the MPR rate was 50%, 40% and 32% with pembrolizumab plus vibostolimab (an anti-TIGIT antibody), gebasaxturev (an oncolytic enterovirus) and MK-4830 (an anti-ILT4 antibody), respectively; 58% with pembrolizumab co-formulated with favezelimab (an anti-LAG-3 antibody); and 47% with pembrolizumab monotherapy. Grade 3 or higher TRAEs occurred in 8%, 28%, 16%, 15% and 7% of patients, respectively^14^. The duration and drug exposure during neoadjuvant therapy or the longer time to resection (as opposed to drug exposure) may impact MPR, offering a longer timeframe for patients to generate an effective antitumor immune response. In our study, pRR was evaluated at the earliest feasible timepoint (6 weeks after the first neoadjuvant treatment was administered). In some of the aforementioned studies, pathological assessment was performed later versus in our study, potentially confounding the efficacy results and comparisons across studies. Based on the safety profile of tobemstomig in our study and reported in the literature, the administration of an additional cycle of treatment may have been well tolerated and further increased the observed efficacy.

Previous reports showed that baseline tumor IFNγ score combined with TMB improved the identification of pathological responders versus non-responders in patients with stage III melanoma treated with neoadjuvant nivolumab plus ipilimumab, when compared to either biomarker alone^15^. Similar results were reported in advanced melanoma^16^. IFNγ alone appeared to be the most robust baseline biomarker for EFS across different cohorts in the PRADO study^17^. We identified additional baseline immune features in the TME associated with pathological response, including CD3^+^ and CD8^+^ TIL density, CD8 T_eff_ cells and *PDCD1* gene expression that correlated with the IFNγ gene signature. The addition of TMB improved the predictive capacity of these biomarkers; however, our data also suggest that they perform robustly individually, which may simplify patient selection.

The baseline biomarkers associated with pathological response were consistent across treatments, suggesting that they may be driven by the common mechanism of PD-1/PD-L1 blockade. These associations likely identify patients predisposed to respond to any CPI. However, because we also observed differences in drug MOA based on immune cell dynamics in the TME and efficacy, some patients may respond preferentially to specific therapies. Identifying treatment-specific biomarkers for immunotherapy remains a major challenge. Given that only a subset of patients were potentially treatment-specific responders in our study, the associations reported are primarily driven by those susceptible to any CPI. Our study lacks sufficient patient numbers and statistical power to effectively distinguish such treatment-specific biomarkers. Additionally, there was an imbalance between arms, with patients in the nivolumab plus ipilimumab arm having a more inflamed TME at baseline versus other arms. This baseline imbalance, coupled with the low sample size, may further limit our ability to identify treatment-specific predictive biomarkers. Finally, bulk RNA sequencing (RNA-seq) has limited resolution to assess specific cell types and their functional states, which may underlie differential responses to the inhibition of specific immune checkpoints. Identifying treatment-specific responders may require profiling at the level of individual cell types, necessitating the use of single-cell RNA-seq in sufficiently large cohorts. Future biomarker studies to identify patients benefiting preferentially from specific CPI treatments are needed.

Tobemstomig tended to increase the ratio of CD8 to FOXP3 in the TME, whereas this increase was not apparent with nivolumab plus ipilimumab treatment, suggesting a potential mechanistic differentiation of treatments. These observations were in line with the avidity-driven selectivity gain of tobemstomig to PD-1/LAG-3 co-expressing T cells over T_reg_ cells^11^. Similar observations were made in the first-in-human trial of tobemstomig (manuscript in progress). Functional studies are needed to further elucidate the role of T_reg_ cells in mediating efficacy.

The most profound changes in immune cell dynamics within the TME were aligned with the observed efficacy outcomes, with the most substantial effects seen in the treatment arms demonstrating the greatest clinical benefit, namely tobemstomig and nivolumab plus ipilimumab. The addition of tiragolumab to tobemstomig or atezolizumab did not enhance immune cell activity in the TME, also consistent with corresponding efficacy data. Whole-transcriptome analysis revealed no significant differences in immune cell dynamics between tobemstomig monotherapy and tobemstomig plus tiragolumab combination, including in MOA-related genes or activation-induced cell death pathways. In a pooled analysis, there was a trend for greater tumor-infiltrating T cell dynamics in pathological responders versus non-responders.

In line with previous reports^10,18^, radiographic response underestimated pathological response in some patients in our study, underlining pathological response as the gold standard in this setting^19,20^. Use of a ctDNA assay (personalized to patient-specific tumor somatic mutations) facilitated a high detection rate in this study (90%) compared to previous reports in patients with early-stage melanoma (approximately 40%)^21–23^. Chan et al.^21^ reported clearance pre-surgery in seven of nine patients whose disease did not recur in the OpACIN-neo and PRADO studies, with positive ctDNA post-surgery, irrespective of pre-treatment ctDNA status, predictive of recurrence. Ultrasensitive ctDNA detection pre-surgery has also been shown to be highly prognostic for overall survival in early-stage lung cancer^24^. Using a high-sensitivity ctDNA detection methodology, we demonstrated that a significant proportion of patients with pCR clear ctDNA as early as after one treatment cycle, a finding consistent with profound decreases in viable tumor in on-treatment biopsies from the same patients at the same timepoint. Our findings support the utility of ctDNA monitoring pre-surgery to inform clinical decision-making on (neo)adjuvant treatment or surgery de-escalation and warrant confirmatory prospective studies that link ctDNA dynamics to long-term endpoints.

This study has several limitations. Beyond region and baseline lactate dehydrogenase (LDH), no other stratification factors were used at randomization due to the small study population, resulting in some imbalances between groups (for example, for baseline CD8 and PD-L1 status), which is common in small, signal-seeking studies. Adjuvant treatment was not mandated but was left to the discretion of the investigator, and patients were followed for a minimum of 12 weeks only, due to closure of the study. Consequently, EFS, relapse-free survival (RFS) and overall survival were not considered meaningful endpoints and were not reported. Correlative analysis of biomarkers and pathological response was limited by sample size; in particular, few pNR patients were evaluated in the nivolumab plus ipilimumab arm. At TLND, it was not feasible to collect and evaluate biomarkers due to limited availability of tumor tissue (especially from responding patients), restricting our ability to assess if the immune dynamics after a single treatment cycle were sustained, expanded or contracted with the subsequent treatment cycle. In addition, bulk RNA-seq analysis did not enable precise characterization of T cell functional states correlated with response or induced by treatment. Single-cell RNA-seq efforts are underway to gain deeper insight into T cell subset biology, in particular stem-like T cells and their progeny. Finally, the study did not fully evaluate the potential of tiragolumab to deepen and prolong clinical response, as RFS, EFS and overall survival were not assessed.

Morpheus-Melanoma clinically validated the MOA of tobemstomig, a bispecific that selectively engages PD-1/LAG-3 co-expressing TILs over LAG-3^+^ T_reg_ cells. Tobemstomig induced robust immune activation, deep pathological responses and favorable safety in stage III melanoma, providing early clinical support for this bispecific strategy. However, further development of tobemstomig has been discontinued based on results from multiple randomized phase 2 trials across several tumor types, including NSCLC (NCT05775289)^25^, esophageal squamous cell carcinoma (ESCC, NCT04785820) and renal cell carcinoma (NCT05805501). In these trials, tobemstomig monotherapy or combination regimens did not show clear benefit over standard-of-care regimens containing anti-PD1 (for example, pembrolizumab or nivolumab) (unpublished data on file; sponsor), suggesting that tobemstomig was unlikely to provide sufficient added benefit to support a broad development strategy as a new CPI across solid tumors.

Nevertheless, the trial confirms the feasibility and potential utility of bispecific immune CPIs in early-stage disease, even in the absence of a broader label. The efficacy and safety profile observed suggest that development of dual PD-1/LAG-3 targeting strategies may still be warranted in selected indications, particularly melanoma, where LAG-3 biology may be uniquely relevant. Additionally, identification of robust baseline and on-treatment biomarkers, including immune gene signatures, TIL density and ctDNA clearance, provides a path forward for patient enrichment and treatment stratification. Notably, biomarkers were associated with response across treatment arms, suggesting broader applicability across CPI and immuno-oncology combinations. These findings can help inform personalized neoadjuvant treatment approaches aimed at maximizing efficacy while minimizing toxicity. These data also add to the growing comparative landscape of neoadjuvant immunotherapy in melanoma, which could help prioritize future dual CPI strategies. Going forward, deeper mechanistic investigation will be needed to understand why dual PD1/LAG-3 blockade appears more effective in melanoma versus other tumor types. A cross-indication biomarker analysis is underway to compare the immune microenvironments of responsive and non-responsive tumors (for example, melanoma versus NSCLC/ESCC), with the aim of elucidating determinants of heterogeneity in patient response to dual PD-1/LAG-3 blockade. The unique MOA of tobemstomig and the comprehensive translational profiling reported here provide a valuable benchmark for such efforts.

In conclusion, in the Morpheus-Melanoma study, tobemstomig demonstrated a similar pRR and improved safety profile compared to nivolumab plus ipilimumab in patients with resectable stage III melanoma. Biomarker analyses identified baseline tumor and longitudinal ctDNA correlates of pathological response that were conserved across treatments and could inform individual risk-adapted treatment. Future studies are needed to refine and mechanistically differentiate CPI combinations using double and triple blocking approaches and to follow patients for long-term survival.

## Methods

### Study design and participants

Morpheus-Melanoma was a global, phase 1b/2, open-label, multicenter, randomized umbrella study conducted at 14 centers across Australia, France, Italy, Spain and the United States with two cohorts: stage III melanoma (cohort 1) and stage IV melanoma (cohort 2). The trial was designed with the flexibility to open new treatment arms as novel treatment options became available and close existing treatment arms that demonstrated minimal clinical efficacy or unacceptable toxicity. For novel combinations that were tested clinically for the first time in this study, a minimum of six patients with previously treated stage IV melanoma were enrolled into the safety run-in phase (cohort 2). If the treatment was determined to be tolerable during the safety run-in phase, the same treatment arm opened for enrollment (cohort 1). Tolerability was determined if less than 30% of a minimum of six patients experienced a grade 3 or higher TRAE that did not improve to grade 2 or better within 2 weeks; a serious TRAE; a TRAE that required permanent discontinuation of study drug; or death, except those that have been incontrovertibly related to disease progression or extraneous causes.

For cohort 1, eligible patients were randomized to one of several treatment arms or the control arm. Enrollment within the experimental arms took place in two phases: a preliminary phase followed by an expansion phase. Approximately 20 patients per arm were enrolled in the preliminary phase, with the potential to add 20 additional patients in the expansion phase if meaningful clinical activity was observed during the preliminary phase.

The assessment of sex was conducted without a predefined methodology and was used for descriptive purposes only. Investigators reported patients’ sex based on local procedures. Gender was not assessed or reported in this study. The reporting of race was optional and reported only in countries where local regulations permitted such documentation.

We report data only from cohort 1—that is, patients with resectable stage III melanoma who have been treated in the neoadjuvant setting.

Eligible patients were 18 years of age or older with resectable stage III melanoma with measurable lymph node metastases (per RECIST version 1.1) that could be biopsied and had an ECOG performance status of 0 or 1. Patients were excluded from the trial if they had a history of in-transit metastases within the last 6 months or had received prior radiotherapy or systemic cancer therapy for their disease. Full inclusion and exclusion criteria are as follows.

### Inclusion criteria

Patients must have met all of the following criteria to qualify:

- Signed informed consent form
- Aged 18 years or older at the time of signing the informed consent form
- ECOG performance status of 0 or 1
- Ability to comply with the protocol, in the investigator’s judgment
- Histologically confirmed resectable stage III melanoma (T: T0, Tx or T1–4; N: cN1–3 or pN1b/2b/3b; M: M0 according to the American Joint Committee on Cancer, 8th Edition (AJCC-8)^26^ and no history of in-transit metastases within the last 6 months)
- Patients may have presented with primary melanoma with concurrent regional nodal metastasis or a history of primary melanoma or unknown primary melanoma with clinically detected regional nodal recurrence and may have belonged to any of the following groups:
  - ∘ Primary cutaneous melanoma with concurrent clinically/radiologically apparent regional lymph node metastases
  - ∘ Clinically/radiologically detected recurrent melanoma at the proximal regional lymph node(s) basin
  - ∘ Clinically/radiologically detected nodal melanoma (if single site) arising from an unknown primary
- Fit and planned for TLND (as assessed by the surgeon prior to randomization according to local guidelines)
- Measurable disease (at least one target lesion) according to RECIST version 1.1
  - ∘ At least one macroscopic lymph node metastasis (measurable according to RECIST version 1.1) to be biopsied
- Availability of a representative tumor specimen that is suitable for biomarker testing via central laboratory
  - ∘ Baseline tumor tissue samples were collected from all patients by biopsy of a metastatic lymph node at screening.
  - ∘ In addition, archival primary tumor tissue was submitted from all patients. In exceptional cases where no archival primary tissue was available (for example, for patients with unknown primary tumor), enrollment was permitted. For archival tissue, a formalin-fixed, paraffin-embedded (FFPE) tumor specimen in a paraffin block (preferred) with sufficient size and tumor content representation, preferably including the invasive margin or, if available, at least 16 slides containing unstained, freshly cut, serial sections, was submitted along with an associated pathology report.
- Adequate hematologic and end-organ function, defined by the following laboratory test results, obtained within 14 days prior to initiation of study treatment:
  - ∘ Absolute neutrophil count ≥1.5 × 10^9^ per liter (1,500 per microliter)
  - ∘ Lymphocyte count ≥0.5 × 10^9^ cells per liter (500 per microliter)
- Borderline machine lymphocyte counts may have been confirmed by a manual count.
  - ∘ Platelet count ≥100 × 10^9^ per liter (100,000 per microliter)
  - ∘ Hemoglobin ≥90 g l^−1^ (9 g dl^−1^)
  - ∘ Aspartate transferase, alanine aminotransferase and alkaline phosphatase ≤2.5× upper limit of normal (ULN)
  - ∘ Total bilirubin ≤1.5× ULN, with the following exception:
    - ▪ Patients with known Gilbert disease: bilirubin level ≤3× ULN
  - ∘ Creatinine ≤1.5× ULN or creatinine clearance ≥30 ml min^−1^ (calculated using the Cockcroft–Gault formula)
  - ∘ Serum albumin ≥25 g l^−1^ (2.5 g dl^−1^)
  - ∘ For patients not receiving therapeutic anticoagulation: international normalized ratio and activated partial thromboplastin time ≤1.5× ULN
- For patients receiving therapeutic anticoagulation: stable anticoagulant regimen (that is, no new thrombosis, thromboembolic event or bleeding episode within 3 months prior to study treatment start)
- Negative HIV test at screening, with the following exception: Patients with a positive HIV test at screening were eligible provided they were stable on antiretroviral therapy, had a CD4 count ≥200 per microliter and had an undetectable viral load.
  - ∘ Patients without a prior positive HIV test result underwent an HIV test at screening, unless not permitted per local regulations.
- Negative hepatitis B surface antibody and negative total hepatitis B core antibody (HBcAb) test at screening. If a patient had a negative hepatitis B surface antigen test and a positive total HBcAb test at screening, a hepatitis B virus (HBV) DNA test was also performed to rule out active HBV.
- Negative hepatitis C virus (HCV) antibody test at screening or positive HCV antibody test followed by a negative HCV RNA test at screening
  - ∘ The HCV RNA test was performed only for patients who had a positive HCV antibody test.
- For women of childbearing potential: agreement to remain abstinent (refrain from heterosexual intercourse) or use contraceptive measures
- For men: agreement to remain abstinent (refrain from heterosexual intercourse) or use contraceptive measures and agreement to refrain from donating sperm

### Exclusion criteria

Patients who met any of the following criteria were excluded from study entry:

- Mucosal and uveal melanoma
  - ∘ Acral lentiginous melanoma is excluded.
- Distantly metastasized melanoma
- History of in-transit metastases within the last 6 months
- Prior radiotherapy
- Prior immunotherapy, including anti-CTLA-4, anti-PD-1 and anti-PD-L1 therapeutic antibodies, and other systemic therapy for melanoma
- Treatment with investigational therapy within 28 days prior to initiation of study treatment
- Treatment with systemic immunostimulatory agents (including, but not limited to, IFN and interleukin-2) within 4 weeks or five drug-elimination half-lives (whichever is longer) prior to initiation of study treatment
- Prior allogeneic stem cell or solid organ transplantation
- Known immunodeficiency or conditions requiring treatment with systemic immunosuppressive medication (including, but not limited to, cyclophosphamide, azathioprine, methotrexate, thalidomide and anti-TNF agents) or anticipation of need for systemic immunosuppressant medication during study treatment, with the following exceptions:
  - ∘ Patients on replacement doses of corticosteroids to manage hypopituitary or adrenal insufficiency are eligible for the study.
  - ∘ Patients who received acute, low-dose, systemic immunosuppressant medications or a one-time pulse dose of systemic immunosuppressant medication (for example, 48 hours of corticosteroids for a contrast allergy) were eligible for the study. Patients requiring chronic low-dose systemic corticosteroid treatment (that is, a maximal dose of corticosteroids ≤10 mg d^−1^ equivalent prednisone) were eligible.
  - ∘ Patients who received mineralocorticoids (for example, fludrocortisone), corticosteroids for chronic obstructive pulmonary disease or asthma or low-dose corticosteroids for orthostatic hypotension or adrenal insufficiency were eligible for the study.
- Treatment with a live, attenuated vaccine within 4 weeks prior to initiation of study treatment or anticipation of need for such a vaccine during study treatment or within 5 months after the final dose of study treatment
- Active or history of autoimmune disease or immune deficiency, including, but not limited to, myasthenia gravis, myositis, autoimmune hepatitis, systemic lupus erythematosus, rheumatoid arthritis, inflammatory bowel disease, antiphospholipid antibody syndrome, Wegener granulomatosis, Sjögren syndrome, Guillain–Barré syndrome or multiple sclerosis, with the following exceptions:
  - ∘ Patients with a history of autoimmune-related hypothyroidism who were on thyroid replacement hormone were eligible for the study.
  - ∘ Patients with controlled type 1 diabetes mellitus who were on a stable insulin regimen were eligible for the study.
  - ∘ Patients with eczema, psoriasis, lichen simplex chronicus or vitiligo with dermatologic manifestations only (for example, patients with psoriatic arthritis were excluded) were eligible for the study provided all of following conditions were met:
    - ▪ Rash covered less than 10% of body surface area.
    - ▪ Disease was well controlled at baseline and required only low-potency topical corticosteroids.
    - ▪ There was no occurrence of acute exacerbations of the underlying condition requiring psoralen plus ultraviolet A radiation, methotrexate, retinoids, biologic agents, oral calcineurin inhibitors or high-potency or oral corticosteroids within the previous 12 months.
- History of idiopathic pulmonary fibrosis, organizing pneumonia (for example, bronchiolitis obliterans), drug-induced pneumonitis or idiopathic pneumonitis or evidence of active pneumonitis on screening chest computed tomography scan. Patients with a history of cancer immunotherapy-related pneumonitis lower than grade 2 were eligible.
- History of malignancy other than malignant melanoma within 2 years prior to screening, with the exception of malignancies with a negligible risk of metastasis or death (for example, 5-year overall survival rate >90%), such as adequately treated carcinoma in situ of the cervix, non-melanoma skin carcinoma, localized prostate cancer, ductal carcinoma in situ or stage I uterine cancer
- Active tuberculosis
- Severe infection within 4 weeks prior to initiation of study treatment, including, but not limited to, hospitalization for complications of infection, bacteremia or severe pneumonia or any active infection that, in the opinion of the investigator, could impact patient safety
- Treatment with therapeutic or prophylactic oral or intravenous antibiotics within 2 weeks prior to initiation of study treatment
- Significant cardiovascular disease, such as New York Heart Association cardiac disease (class II or higher), myocardial infarction or cerebrovascular accident within 3 months prior to initiation of study treatment, unstable arrhythmia or unstable angina
- Uncontrolled hypertension (defined as resting systolic blood pressure >150 mmHg and/or diastolic blood pressure >100 mmHg in two or more serial measurements)
- Major surgical procedure, other than for diagnosis, within 4 weeks prior to initiation of study treatment or anticipation of need for a major surgical procedure other than TLND during the study
  - ∘ Placement of central venous access catheter (for example, port or similar) was not considered a major surgical procedure and was, therefore, permitted.
- Any other disease, metabolic dysfunction, physical examination finding or clinical laboratory finding that contraindicated the use of an investigational drug, may have affected the interpretation of the results, impaired the ability of the patient to participate in the study or may have rendered the patient at high risk from treatment complications
- History of severe allergic reactions to chimeric or humanized antibodies or fusion proteins
- Known hypersensitivity to Chinese hamster ovary cell products or recombinant human antibodies
- Known allergy or hypersensitivity to any of the study drugs or their excipients
- Known intolerance to any of the drugs required for premedication (acetaminophen, ranitidine, diphenhydramine and methylprednisolone)
- Pregnancy or breastfeeding or intention of becoming pregnant during the study
  - ∘ Women of childbearing potential must have had a negative serum pregnancy test result within 14 days prior to initiation of study treatment.
- Eligible only for the control arm

Patients who met any of the following criteria were excluded from the tobemstomig-containing arms:

- Prior treatment with an anti-LAG-3 agent
- History of myocarditis (regardless of etiology)
- Left ventricular ejection fraction less than 50% assessed by either transthoracic echocardiogram (TTE) or multiple-gated acquisition scan (TTE preferred test) within 6 months prior to initiation of study treatment
- Troponin T (TnT) or troponin I (TnI) > institutional ULN
- Patients with TnT or TnI levels between >1 and <2× ULN were eligible if repeat levels within 24 hours were ≤1× ULN. If repeat levels within 24 hours were between >1 and <2× ULN, patients needed to undergo a cardiac evaluation and may have been considered for treatment if there were no clinically significant findings.

Patients who met any of the following criteria were excluded from the tiragolumab-containing arms:

- Prior treatment with an anti-TIGIT agent
- Acute Epstein–Barr virus (EBV) infection or known or suspected chronic active EBV infection at screening

Patients with a positive EBV viral capsid antigen IgM test at screening were excluded from this arm. An EBV polymerase chain reaction (PCR) test was performed as clinically indicated to screen for active infection or suspected chronic active infection. Patients with a positive EBV PCR test were excluded from this arm.

The study protocol was approved by the institutional review board at each participating center and complied with Good Clinical Practice guidelines, the principles of the Declaration of Helsinki and local laws. An independent data monitoring committee was in place, and all patients provided written informed consent.

### Randomization and masking

The study employed a permuted-block randomization method with dynamically changing randomization ratios to account for the fluctuation in the number of experimental treatment arms that were open for enrollment during the study. The control arm remained open throughout the study with the stipulation that the likelihood of being allocated to the control arm was no more than 35%. Eligible patients were randomly assigned to each treatment arm using an interactive web-based response system. Randomization was stratified according to geographic region and baseline LDH.

### Procedures

Eligible patients were randomized to receive one of the following treatments every 3 weeks for 6 weeks in the neoadjuvant setting (that is, two doses): tobemstomig 2,100 mg; tobemstomig 2,100 mg plus tiragolumab 600 mg; atezolizumab 1,200 mg plus tiragolumab 600 mg; or nivolumab 3 mg kg^−1^ plus ipilimumab 1 mg kg^−1^. TLND occurred at week 7, and pathological response was evaluated according to International Neoadjuvant Melanoma Consortium (INMC) criteria^26^.

### Outcomes

The primary efficacy endpoint was pRR as assessed by independent pathological review at the time of surgery, defined as the proportion of patients with pCR, npCR and pPR. For CPI treatment, pathological response is a well-established surrogate endpoint for RFS^6,19^. Secondary efficacy endpoints were pRR by local pathological assessment and investigator-assessed ORR per RECIST version 1.1 (defined as the proportion of patients with a complete response or a partial response prior to surgery). Safety was an endpoint, and biomarkers were an exploratory endpoint.

### Tumor and blood collection for exploratory biomarker analysis

Tumor biopsies were obtained from lymph node metastasis (3–4 core biopsies, 14-gauge needle) prior to treatment and at week 3 (pre-dose) and were formalin fixed and paraffin embedded. Blood was collected for biomarker evaluation pre-treatment, at week 3, at the time of surgery (week 7) and postoperatively at weeks 10 and 13.

### Histology

FFPE tumor tissue blocks were obtained and sectioned consecutively for hematoxylin and eosin (H&E), immunohistochemistry (IHC) and/or immunofluorescence stains, which were performed at either Discovery Life Sciences (Kassel, Germany) or Roche Tissue Diagnostics (Tucson, Arizona) laboratories. Samples passing quality control for sufficient tumor content without necrotic areas and excluding lymph node tissue contamination were included in the analysis. Details regarding the IHC and immunofluorescence assays performed can be found in Supplementary Table 4.

### DNA and RNA isolation

FFPE tumor tissues were macro-dissected from normal tissue and subjected to DNA and RNA co-extraction using an AllPrep DNA/RNA FFPE Kit (Qiagen), following the manufacturer’s protocol in a QIAcube (Qiagen). RNA was assessed by a 2100 Bioanalyzer (Agilent). Genomic DNA (gDNA) was extracted from blood using a Puregene DNA Purification Kit (Gentra Systems).

### RNA-seq

Bulk RNA-seq was performed with a TruSeq RNA Exome Kit (Illumina), which captures the coding transcriptome and is optimized for sequencing RNA from FFPE tissues. Pre-hybridization and post-hybridization capture molecules were amplified using PCR, with 15 cycles each. Final libraries were assessed using quantitative PCR for quantitation and TapeStation for fragment size assessment. Libraries were sequenced on a NovaSeq 6000 (Illumina) using the Xp workflow using 50 base pairs paired-end sequencing and a minimum read depth of 40 million reads per sample. The RNA-seq data processing involved mapping the reads to the human reference genome (hg38) using the STAR algorithm^27^. Gene-level read counts were collated and normalized to account for variations in library size and composition. The analysis was restricted to genes expressed in at least three samples, with a minimum read count per million greater than 1. Differential gene expression analysis was conducted using the voom/limma framework^28^. To identify enriched gene signatures (Supplementary Table 5) and pathways among the differentially expressed genes, we applied the CAMERA method, a competitive gene set test that considers inter-gene correlations^29^. We defined the entire set of protein-coding genes as the background. CAMERA was then employed to analyze the ranked differentially expressed genes for each contrast, using the standard inter-gene correlation factor of 0.01.

### Whole-exome sequencing

Whole-exome sequencing (WES) was performed on DNA extracted from macro-dissected FFPE tumor tissue and gDNA from matched normal blood samples as described^30^. Paired-end FASTQ files from normal and tumor samples were mapped to the human genome (hg38) using BWA (version 0.7.17). Unmapped reads were removed, and mapped reads were sorted and duplicates removed using SAMtools and Picard’s MarkDuplicates, respectively. Base quality score recalibration was performed using GATK (version 4.5.0.0)^31^. Somatic mutations were identified using GATK Mutect2 (version 4.1.8.1) in paired mode and annotated with VEP (version 108.0)^32^. For TMB computation, somatic mutations were filtered based on criteria including tumor allele frequency (>10%), read depth (>10 reads), population frequency (<5% in the Genome Aggregation Database)^33^, gene location (protein-coding genes), mutation type (missense, nonsense and nonstop mutations) and mappability (excluding low mappability regions defined by Amemiya et al.^34^). The total count of somatic mutations per patient, after applying these filters, was normalized by the length of the target exonic region (34 megabases) to estimate TMB in units of mutations per megabase.

### Composite scores integrating immune biomarkers and TMB

Composite scores integrating pre-treatment immune biomarker profiles with TMB were generated using a robust, non-parametric rank-based method. For each patient, two independent rankings were computed: (1) a percentile rank based on their baseline immune biomarker level (for example, PD-L1 expression) and (2) a percentile rank based on their TMB value. Percentile normalization (0–100 scale) ensured equal weighting of biomarkers and TMB, regardless of their absolute scales or dynamic ranges. The final composite score for each patient was derived as the arithmetic mean of their normalized biomarker and TMB percentiles. Higher composite scores reflect concurrent elevation in both immune biomarker levels and TMB. These scores were treated as continuous variables in downstream analyses to evaluate their association with response.

### ctDNA analysis

A personalized, tumor-informed, 16-plex PCR next-generation sequencing assay (Signatera RUO; Natera) was used for the longitudinal detection and quantification of ctDNA. In brief, a set of 2–16 patient-specific somatic single-nucleotide variants (SNVs) from WES results was selected for multiplex PCR (mPCR). The mPCR primers targeting the personalized SNVs were used to track ctDNA in the corresponding patients’ plasma samples. Cell-free DNA was extracted from patient plasma (median, 4.6 ml; range, 1.1–5.5 ml) at a given timepoint and was used to detect ctDNA. Plasma samples with two or more SNVs detected were defined as ctDNA positive. ctDNA concentration was reported in mean tumor molecules per milliliter of plasma^30^.

### Statistical analysis of clinical and safety endpoints

This study was designed to obtain preliminary efficacy, safety and biomarker data on immunotherapy-based treatment combinations when administered to patients with melanoma. The study was not designed with explicit power and type I error considerations for a hypothesis test. In a preliminary phase, a sample size of approximately 20 patients for the experimental arms in cohort 1 (tobemstomig, tobemstomig plus tiragolumab and atezolizumab plus tiragolumab) was considered sufficient to obtain preliminary efficacy and safety data on treatments or treatment combinations. If promising results were observed in an interim analysis of an experimental arm, the study had the potential to add 20 additional patients in the expansion phase.

Expansion of an experimental arm was gated on observing clinically meaningful pRR in the treatment arm relative to the internal control arm. Decisions regarding further development of a treatment combination were informed by calculating the Bayesian posterior probability of equal pRR between experimental and control arms. If the posterior probability that the pRR was similar was sufficiently high (for example, >50%), the respective arm could be opened for enrollment of an additional 20 patients. The final decision-making for expansion considered the benefit–risk balance of the totality of the data, including safety data as well as other potential emerging external information.

The efficacy-evaluable population was defined as all patients who received at least one dose of each drug for their treatment. The safety-evaluable population was defined as all patients who received any study treatment. Demographic and baseline characteristics were summarized using descriptive statistics. pRR, MPR and ORR were calculated for each arm along with 95% confidence intervals (Clopper–Pearson method). Adverse events were summarized and mapped to the Medical Dictionary for Regulatory Activities thesaurus terms. Immune-mediated adverse events were summarized by medical concept, and TLND delays due to TRAEs were analyzed. Due to limited sample size, no stratified analysis was performed, and randomization stratification by geographic region and baseline LDH was only applied to facilitate balance for those factors. No statistical analysis based on sex was performed as it was used for descriptive purposes only. All analyses were done using SAS version 9.4 software.

### Reporting summary

Further information on research design is available in the Nature Portfolio Reporting Summary linked to this article.

## Online content

Any methods, additional references, Nature Portfolio reporting summaries, source data, extended data, supplementary information, acknowledgements, peer review information; details of author contributions and competing interests; and statements of data and code availability are available at 10.1038/s41591-025-03967-2.

## Supplementary information

Supplementary Information Supplementary Tables 1–4, Supplementary Figs. 1–4 and List of independent ethics committees/institutional review boards

Reporting Summary

## Data Availability

Qualified researchers may request access to individual patient-level clinical and biomarker data through the clinical study data request platform (https://vivli.org/). This trial enrolled across multiple regions, and national data protection interpretations do not enable us to publicly deposit individual patient raw sequencing files. Further details on Roche’s criteria for eligible studies are available at https://vivli.org/members/ourmembers/. For further details on Roche’s Global Policy on the Sharing of Clinical Information and how to request access to related clinical study documents, see https://www.roche.com/research_and_development/who_we_are_how_we_work/clinical_trials/our_commitment_to_data_sharing.htm.
