# Supplementary material for: Neoadjuvant PD-1 and LAG-3-targeting bispecific antibody and other immune checkpoint inhibitor combinations in resectable melanoma: the randomized phase 1b/2 Morpheus-Melanoma trial
Source: Nat Med. 2025 Sep 24;31(11):3700–12. doi: 10.1038/s41591-025-03967-2 (PMC12618235; doi:10.1038/s41591-025-03967-2)
Supplement: Supplementary file 1 — Supplementary Tables 1–4, Supplementary Figs. 1–4 and List of independent ethics committees/institutional review boards [file 41591_2025_3967_MOESM1_ESM.pdf]

nature medicine

Supplementary information

https://doi.org/10.1038/s41591-025-03967-2

Neoadjuvant PD-1 and LAG-3-targeting bispecific antibody and other immune checkpoint inhibitor combinations in resectable melanoma: the randomized phase 1b/2 Morpheus-Melanoma trial

In the format provided by the authors and unedited

Supplementary Materials

Table S1 | Pathological response rate by independent pathological review and local pathological assessment

	Tobemstomig (*n* = 40)	Tobemstomig + Tiragolumab (*n* = 20)	Atezolizumab + Tiragolumab (*n* = 20)	Nivolumab + Ipilimumab (*n* = 22)
**Independent pathological review**				
pRR	32 (80.0%)	12 (60.0%)	9 (45.0%)	17 (77.3%)
95% CI	(64.35, 90.95)	(36.05, 80.88)	(23.06, 68.47)	(54.63, 92.18)
MPR	25 (62.5%)	8 (40.0%)	8 (40.0%)	16 (72.7%)
95% CI	(45.80, 77.27)	(19.12, 63.95)	(19.12, 63.95)	(49.78, 89.27)
pCR	19 (47.5%)	8 (40.0%)	5 (25.0%)	15 (68.2%)
95% CI	(31.51, 63.87)	(19.12, 63.95)	(8.66, 49.10)	(45.13, 86.14)
npCR	6 (15.0%)	0	3 (15.0%)	1 (4.5%)
95% CI	(5.71, 29.84)	(0.00, 16.84)	(3.21, 37.89)	(0.12, 22.84)
pPR	7 (17.5%)	4 (20.0%)	1 (5.0%)	1 (4.5%)
95% CI	(7.34, 32.78)	(5.73, 43.66)	(0.13, 24.87)	(0.12, 22.84)
pNR	5 (12.5%)	6 (30.0%)	9 (45.0%)	4 (18.2%)
95% CI	(4.19, 26.80)	(11.89, 54.28)	(23.06, 68.47)	(5.19, 40.28)
Not evaluable	1 (2.5%)	1 (5.0%)	0	1 (4.5%)
95% CI	(0.06, 13.16)	(0.13, 24.87)	(0.00, 16.84)	(0.12, 22.84)
Missing	2 (5.0%)	1 (5.0%)	2 (10.0%)	0
**Local pathological assessment**				
pRR	30 (75.0%)	12 (60.0%)	10 (50.0%)	18 (81.8%)
95% CI	(58.80, 87.31)	(36.05, 80.88)	(27.20, 72.80)	(59.72, 94.81)
MPR	25 (62.5%)	8 (40.0%)	7 (35.0%)	17 (77.3%)
95% CI	(45.80, 77.27)	(19.12, 63.95)	(15.39, 59.22)	(54.63, 92.18)

pCR	19 (47.5%)	7 (35.0%)	5 (25.0%)	14 (63.6%)
95% CI	(31.51, 63.87)	(15.39, 59.22)	(8.66, 49.10)	(40.66, 82.80)
npCR	6 (15.0%)	1 (5.0%)	2 (10.0%)	3 (13.6%)
95% CI	(5.71, 29.84)	(0.13, 24.87)	(1.23, 31.70)	(2.91, 34.91)
pPR	5 (12.5%)	4 (20.0%)	3 (15.0%)	1 (4.5%)
95% CI	(4.19, 26.80)	(5.73, 43.66)	(3.21, 37.89)	(0.12, 22.84)
pNR	8 (20.0%)	7 (35.0%)	8 (40.0%)	4 (18.2%)
95% CI	(9.05, 35.65)	(15.39, 59.22)	(19.12, 63.95)	(5.19, 40.28)
Not evaluable	0	0	0	0
95% CI	(0.00, 8.81)	(0.00, 16.84)	(0.00, 16.84)	(0.00, 15.44)
Missing	2 (5.0%)	1 (5.0%)	2 (10.0%)	0

95% CIs for rates were constructed using Clopper-Pearson method.
pRR: ≤50% viable tumor cells; MPR: ≤10% viable tumor cells; pCR: 0% viable tumor cells; npCR: >0% to ≤10% viable tumor cells; pPR: >10 to ≤50% viable tumor cells; pNR: > 50% viable tumor cells.
CI, confidence interval; MPR, major pathological response; npCR, near pathological complete response; pCR, pathological complete response; pNR, pathological no response; pPR, pathological partial response.

Table S2 | Time to onset and duration of Grade ≥3 AEs

AE	Tobemstomig (*n* = 40)				Tobemstomig + Tiragolumab (*n* = 20)			
	**Patients with AE, *n* (%)**	**Time to earliest onset, days**	**Duration, days**	**Resolved?**	**Patients with AE, *n* (%)**	**Time to earliest onset, days**	**Duration, days**	**Resolved?**
ALT increased	0	-	-	-	0	-	-	-
Blood cholesterol increased	1 (2.5)	52	≤14	Yes	0	-	-	-
Catheter site infection	1 (2.5)	56	≤14	Yes	0	-	-	-
Cellulitis	1 (2.5)	64	≤14	Yes	1 (5.0)	57	>14	Yes
Diabetic ketoacidosis	0	-	-	-	1 (5.0)	293	≤14	Yes
Hematoma	0	-	-	-	1 (5.0)	53	≤14	Yes
Hepatitis	0	-	-	-	0	-	-	-
Hepatic cytolysis	0	-	-	-	0	-	-	-
Hyperlipasemia	1 (2.5)	28	>14	Yes	0	-	-	-
Hypertransaminasemia	0	-	-	-	1 (5.0)	22	>14	No
Hypertriglyceridemia	1 (2.5)	47	>14	Yes	0	-	-	-
Immune thrombocytopenia	0	-	-	-	0	-	-	-
Immune-mediated hepatitis	0	-	-	-	0	-	-	-
Immune-mediated lung disease	0	-	-	-	0	-	-	-
Infected seroma	1 (2.5)	119	≤14	Yes	0	-	-	-
Lipase increased	1 (2.5)	43	≤14	Yes	0	-	-	-
Lymphatic fistula	0	-	-	-	1 (5.0)	61	>14	Yes
Meningitis aseptic	0	-	-	-	0	-	-	-
Myocardial ischemia	1 (2.5)	42	>14	No	0	-	-	-
Pancreatitis	1 (2.5)	45	>14	Yes	0	-	-	-
Pleural effusion	0	-	-	-	1 (5.0)	45	>14	No
Pneumonitis	0	-	-	-	0	-	-	-
Pneumonia	1 (2.5)	81	≤14	Yes	0	-	-	-
Postoperative wound infection	0	-	-	-	0	-	-	-
Post procedural hematoma	1 (2.5)	50	>14	Yes	0	-	-	-
Pruritus	0	-	-	-	1 (5.0)	1	>14	No
Rash maculo-papular	0	-	-	-	1 (5.0)	1	>14	No
Seroma	0	-	-	-	1 (5.0)	45	>14	Yes
Ventricular fibrillation	1 (2.5)	49	≤14	Yes	0	-	-	-
Wound dehiscence	1 (2.5)	50	>14	Yes	1 (5.0)	82	>14	Yes

AE, adverse event; ALT, alanine transaminase.

Table S2 | Time to onset and duration of Grade ≥3 AEs (continued)

AE	Atezolizumab + Tiragolumab (*n* = 20)				Nivolumab + Ipilimumab (*n* = 22)			
	Patients with AE, *n* (%)	Time to earliest onset, days	Duration, days	Resolved?	Patients with AE, *n* (%)	Time to earliest onset, days	Duration, days	Resolved?
ALT increased	0	-	-	-	1 (3.8)	88	>14	Yes
Blood cholesterol increased	0	-	-	-	0	-	-	-
Catheter site infection	0	-	-	-	0	-	-	-
Cellulitis	0	-	-	-	1 (3.8)	68	>14	Yes
Diabetic ketoacidosis	0	-	-	-	0	-	-	-
Hematoma	0	-	-	-	0	-	-	-
Hepatitis	0	-	-	-	1 (3.8)	20	≤14	Yes
Hepatic cytolysis	0	-	-	-	1 (3.8)	20	>14	Yes
Hyperlipasemia	0	-	-	-	0	-	-	-
Hypertransaminasemia	0	-	-	-	0	-	-	-
Hypertriglyceridemia	0	-	-	-	0	-	-	-
Immune thrombocytopenia	0	-	-	-	1 (3.8)	59	≤14	Yes
Immune-mediated hepatitis	0	-	-	-	1 (3.8)	34	≤14	Yes
Immune-mediated lung disease	0	-	-	-	1 (3.8)	27	>14	Yes
Infected seroma	0	-	-	-	0	-	-	-
Lipase increased	0	-	-	-	0	-	-	-
Lymphatic fistula	0	-	-	-	0	-	-	-
Meningitis aseptic	0	-	-	-	1 (3.8)	28	≤14	Yes
Myocardial ischemia	0	-	-	-	0	-	-	-
Pancreatitis	0	-	-	-	0	-	-	-
Pleural effusion	0	-	-	-	0	-	-	-
Pneumonitis	0	-	-	-	1 (3.8)	32	>14	Yes
Pneumonia	0	-	-	-	0	-	-	-
Postoperative wound infection	1 (5.0)	63	≤14	Yes	0	-	-	-
Post procedural hematoma	0	-	-	-	0	-	-	-
Pruritus	0	-	-	-	0	-	-	-
Rash maculo-papular	0	-	-	-	0	-	-	-
Seroma	0	-	-	-	0	-	-	-
Ventricular fibrillation	0	-	-	-	0	-	-	-
Wound dehiscence	0	-	-	-	1 (3.8)	122	≤14	Yes

AE, adverse event; ALT, alanine transaminase.

Table S3 | Immune-mediated AEs

Immune-mediated AE	Tobemstomig (*n* = 40)	Tobemstomig + Tiragolumab (*n* = 20)	Atezolizumab + Tiragolumab (*n* = 20)	Nivolumab + Ipilimumab (*n* = 22)
Total number of patients with at least one AE	29 (72.5%)	13 (65.0%)	8 (40.0%)	15 (68.2%)
Immune-mediated rash	12 (30.0%)	6 (30.0%)	5 (25.0%)	7 (31.8%)
Immune-mediated hepatitis (diagnosis and lab abnormalities)	7 (17.5%)	3 (15.0%)	1 (5.0%)	8 (36.4%)
Immune-mediated hyperthyroidism	7 (17.5%)	6 (30.0%)	0	4 (18.2%)
Immune-mediated hypothyroidism	6 (15.0%)	1 (5.0%)	1 (5.0%)	2 (9.1%)
Infusion-related reactions	6 (15.0%)	2 (10.0%)	0	2 (9.1%)
Immune-mediated pancreatitis	5 (12.5%)	1 (5.0%)	0	2 (9.1%)
Immune-mediated pneumonitis	1 (2.5%)	0	1 (5.0%)	2 (9.1%)
Immune-mediated myositis	1 (2.5%)	0	1 (5.0%)	1 (4.5%)
Immune-mediated myositis (myositis + rhabdomyolysis)	1 (2.5%)	0	1 (5.0%)	1 (4.5%)
Immune-mediated colitis	1 (2.5%)	0	0	0
Immune-mediated meningitis	0	0	0	1 (4.5%)
Immune-mediated meningoencephalitis	0	0	0	1 (4.5%)
Immune-mediated ocular inflammatory toxicity	0	0	1 (5.0%)	1 (4.5%)
Immune-mediated diabetes mellitus	0	1 (5.0%)	0	0
Immune-mediated hypophysitis	0	1 (5.0%)	0	0
Immune-mediated myocarditis	0	1 (5.0%)	0	0

AE, adverse event.

Table S4 | Histology reagents and methods

Assay	Method	Section thickness (MuM)	Staining device	Scanning device	Scoring	Biomarkers	Clone	Host	Supplier/Catalog	Detection/ Chromogen/ Fluorophore
CD8/Ki67	IHC	3	VENTANA Discovery Ultra IHC/ISH	Ventana iScan HT	Digital Algorithm, proprietary	CD8	SP239	Rabbit monoclonal	Abcam (ab178089)	Discovery Yellow (yellow)
						Ki67	30-9	Rabbit monoclonal	Ventana (790-4286)	Discovery Purple (purple)
PD-L1	IHC	4	VENTANA Benchmark ULTRA IHC/ISH	n.a.	Visual readout	PD-L1	SP263	Rabbit monoclonal	Ventana (790-4905)	Optiview DAB (brown)
LAG-3	IHC	4	VENTANA Benchmark ULTRA IHC/ISH	n.a.	Visual readout	LAG-3	17B4	Mouse monoclonal	Abcam (ab40466)	Optiview DAB (brown)
ARES #2	IF	3	VENTANA Discovery Ultra IHC/ISH	Zeiss AxioScan Z1	Digital Algorithm, HALO (Indica Labs)	CD3	SP162	Rabbit monoclonal	Abcam (ab135372)	Discovery DCC (teal)
						Perforin	SP476	Rabbit monoclonal	Spring (M7764.R)	Discovery FAM (green)
						FOXP3	SP97	Rabbit monoclonal	Abcam (ab99963)	Discovery Rodhamine 6G (yellow)

IF, immunofluorescence; IHC, immunohistochemistry.

Table S5 | Immune-related gene signatures

Gene signature	Gene list
CD8 Teff	*CD8A, GZMA, GZMB, IFNG, EOMES, PRF1, CXCL9, CXCL10, TBX21*
NK cell	*NCR1, LIM2, KIR2DL4, KLRC1, IL18RAP, KLRF1*
Stem-like CD8 T cell	*S1PR5, SLAMF6, CXCR3, S1PR1, ITGB7, CD8A, IL7R, TCF7, PDCD1*
B cell	*CD19, MS4A, TNFRSF13C, VPREB3, PAX5, CR2*
IFN-γ	*IDO1, CXCL10, CXCL9, HLA-DRA, STAT1, IFNG, CCR5, CXCL11, PRF1, GZMA*
Treg	*FOXP3, CCR8, PMCH, CCR4, RTKN2, CTLA4*
Myeloid	*CSF3R, MS4A64 MS4A7, MNDA, C5AR1, FCGR2A, C3AR1, FPR1, LILRB2, HDC, FCGR3B, CCL22*
MHC pathway	*CALR, PSME2, TAP1, TAPBP, WSB1, PSMB9, PDIA3, PSMB8, IRF1, PSME1, CD74, PSME3*
Melanoma	*MIA, TYR, SLC45A2, CDH19, PMEL, SLC24A5, MAGEA6, GJB1, PLP1, PRAME, CAPN3, ERBB3, GPM6B, S100B, PAX3, S100A1, MLANA*

IFN-γ, interferon gamma; MHC, major histocompatibility; NK, natural killer; Treg, regulatory T cells.

Fig. S1 | Baseline biomarkers by treatment

Boxplots showing expression of immunohistochemistry- and immunofluorescence-based biomarkers in pre-treatment tumor samples, including PD-L1 SP263 TC and TAP scores (n=79), LAG-3 area fraction (n=39), CD3+ T cell density (n=77) and CD8+ T cell density in viable tumor (n=70, tumor nests (n=74) and tumor stroma (n=69). Patients are grouped by treatment. Boxplots illustrate the median (central line), interquartile range (box), minima and maxima (whiskers, up to 1.5 times the interquartile range), with data points beyond this limit shown as individual outliers. Individual patients (dots) colored by pathological response.

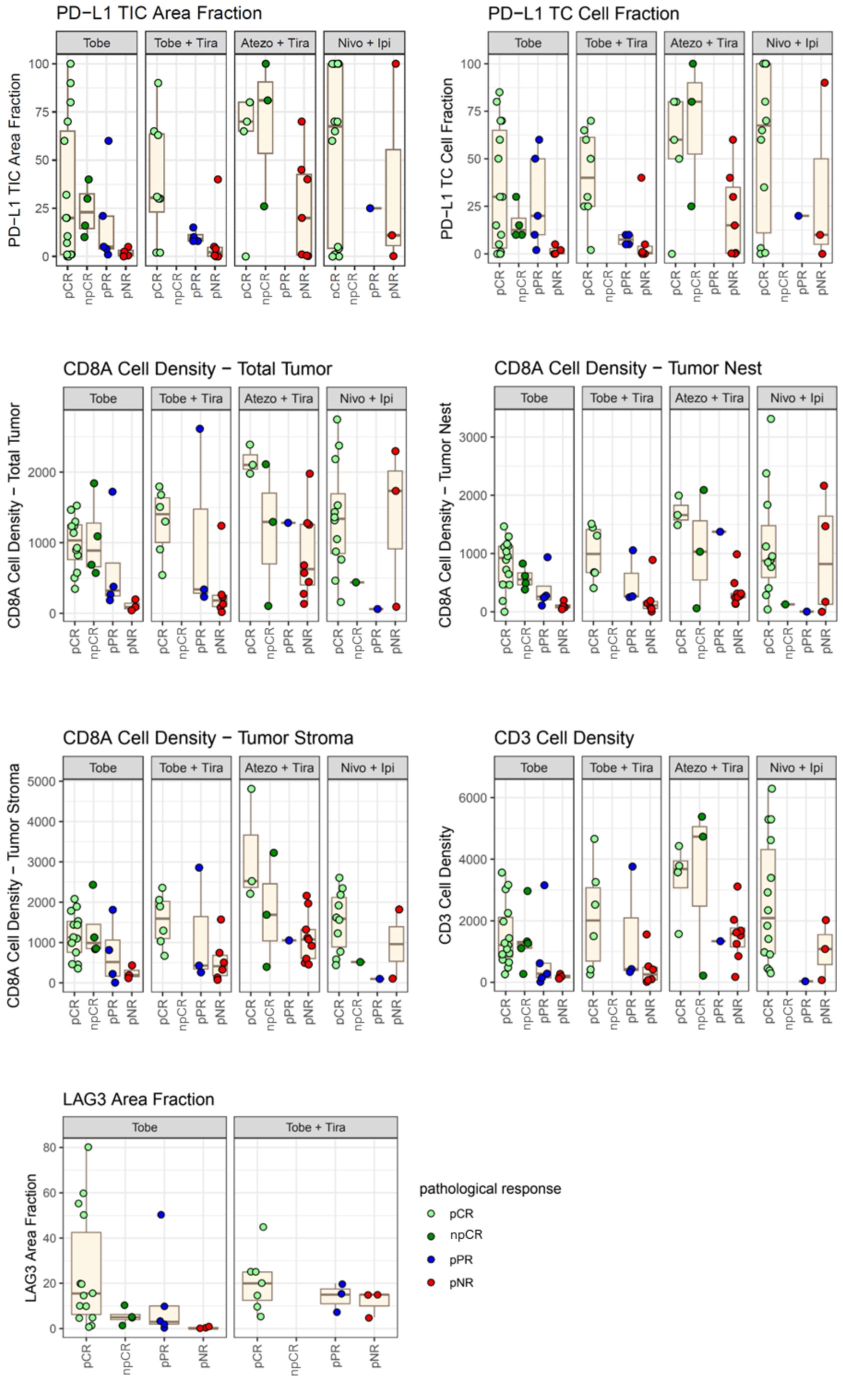

Atezo, atezolizumab; Ipi, ipilimumab; LAG-3, lymphocyte activation gene-3; Nivo, nivolumab; npCR, near pathological complete response; pCR, pathological complete response; PD-L1, programmed death-ligand 1; pNR, pathological no response; pPR, pathological partial response; TAP, tumor area positivity; TC, tumor cell; Tira, tiragolumab; Tobe, tobemstomig

Fig. S2 | Association of baseline tumor microenvironment biomarkers with pathological response

(a) Association between pre-treatment immune-related TME biomarkers and pathological response in patients with available baseline data (n=87). The color gradient represents the area under the receiver operating characteristic curve (AUC, ROC curve), ranging from 0.5 (random prediction) to 1.0 (perfect prediction). Statistical significance determined by two-sided rank-sum test with Benjamini-Hochberg multiple testing correction; significance indicated by asterisks. (b) Bar plots showing the predictive performance (AUC) of select biomarkers for distinguishes pathological responders from non-responders in patients treated with tobemstomig, and the improvement achieved by combining biomarkers with TMB into a composite score.

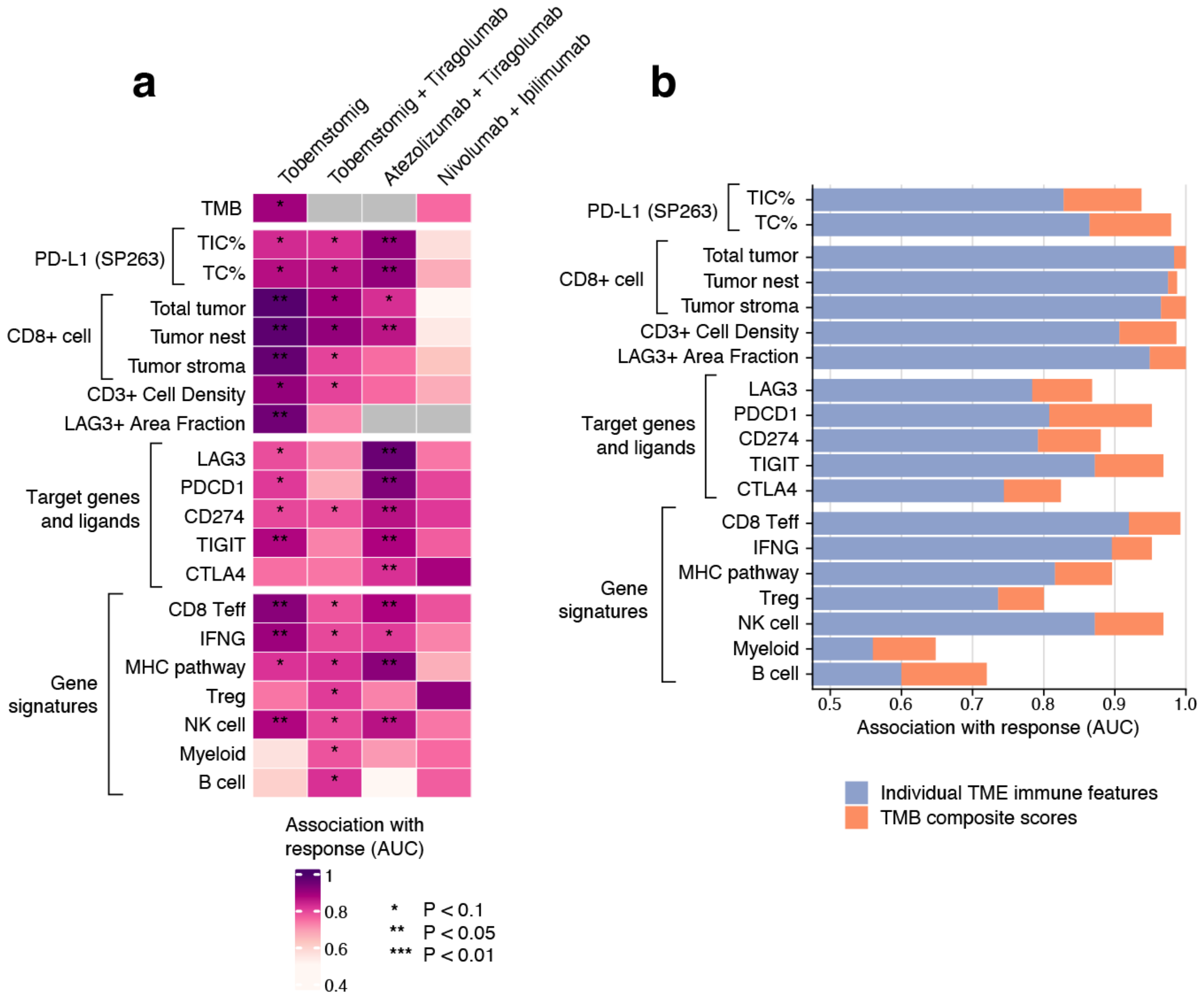

AUC, area under the curve; TMB, tumor mutational burden; TME, tumor microenvironment

Fig. S3 | Immune dynamics in the tumor microenvironment according to treatment for RNA-seq-based biomarkers

Pre- and on-treatment lymph node tumor tissue were evaluated for tumor pharmacodynamics relevant to drug mode of action (n=79). Boxplots showing the change in RNA-seq based immune biomarkers in the tumor microenvironment after one cycle of treatment compared with pre-treatment. Patients are grouped according to treatment. Boxplots illustrate the median (central line), interquartile range (box), minima and maxima (whiskers, up to 1.5 times the interquartile range), with data points beyond this limit shown as individual outliers. Individual patients (dots) colored by pathological response.

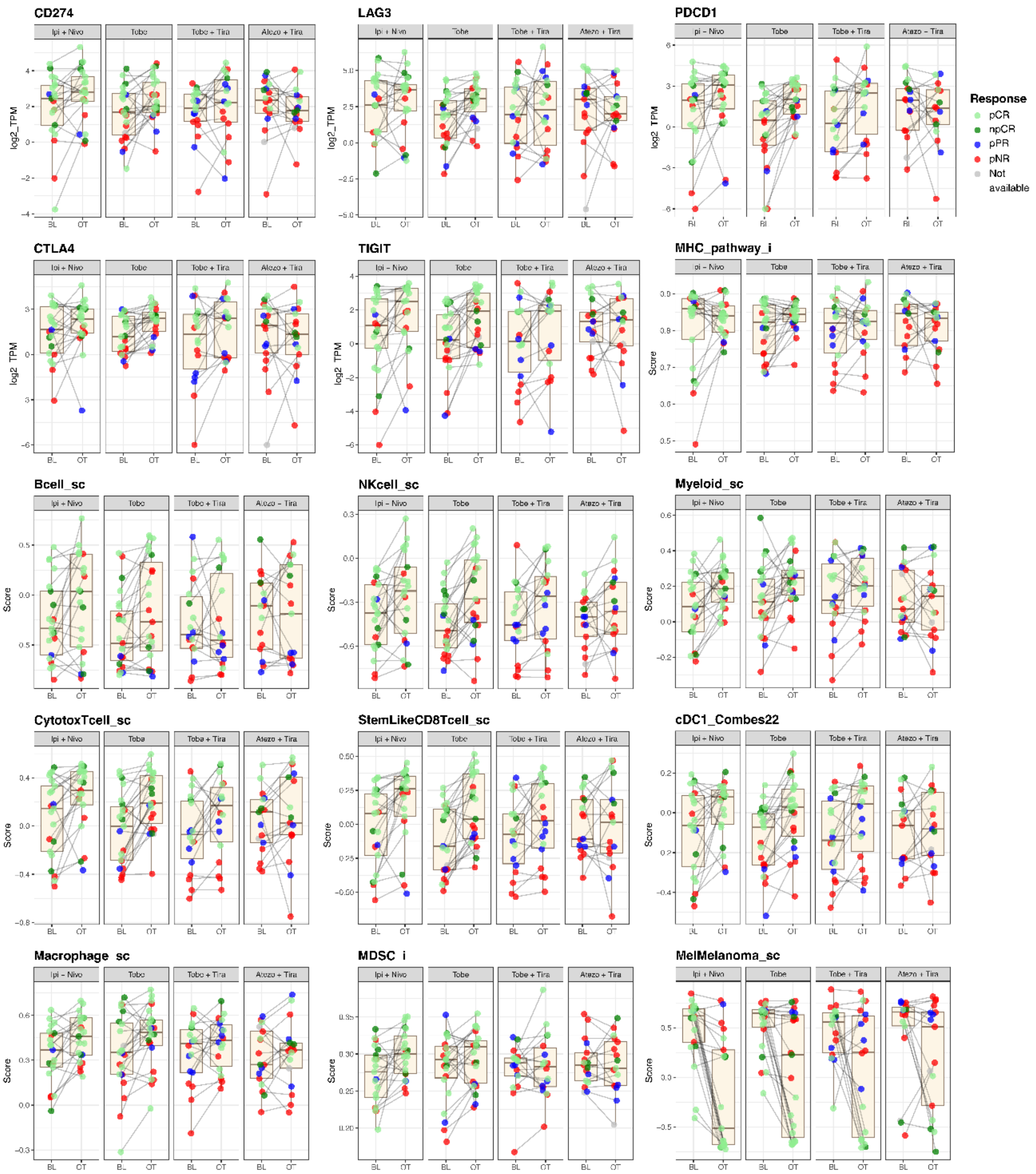

Atezo, atezolizumab; BL, baseline; Ipi, ipilimumab; Nivo, nivolumab; OT, one cycle of treatment; pCR, pathological complete response; pNR, pathological no response; npCR, near pathological complete response; pPR, pathological partial response; RNA-seq, RNA sequencing; Tira, tiragolumab; Tobe, tobemstomig

Fig. S4 | Immune dynamics in the tumor microenvironment by treatment

Pre- and on-treatment lymph node tumor sampleswere analyzedafter one treatment cycle for pharmacodynamic changes in biomarkers relevant to drug mechanism of action (n=xx). Boxplots showing the change in immunohistochemistry and immunofluorescence-based biomarkers, includingnamely PDL1 on tumor cells (n=39), LAG3 area fraction (n=17), CD8+ T cell density in total tumor (n=35), tumor nest (n=36) and tumor stroma (n=57), proliferating CD8 T cells in total tumor (n=35), CD3+ (n=36) and CD3+ Perforin+ (n=36) T cell density, CD3-Perforin+ (n=36) and FOXP3 (n=36) densities and CD8: FOXP3 ratio (n=30). Patients are grouped by to treatment. Boxplots illustrate the median (central line), interquartile range (box), minima and maxima (whiskers, upto 1.5 times the interquartile range), with data points beyond this limit shown as individual outliers. Individual patient values (dots) colored by pathological response. Asterisks indicate significant change from baseline based on two-sided Wilcoxon signed-rank tests (no multiple testing comparison) (*p<0.05, **p<0.01).

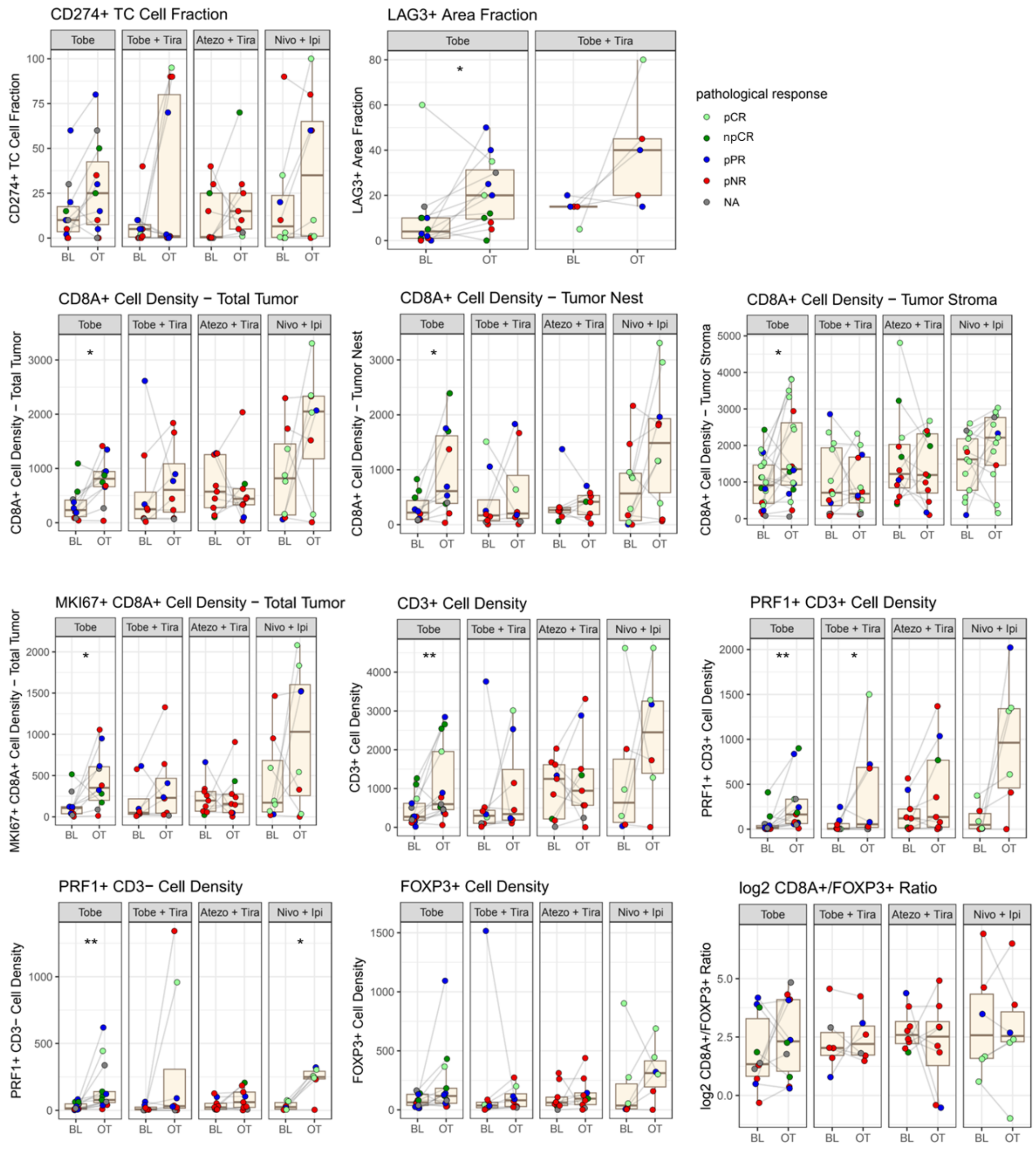

Atezo, atezolizumab; BL, baseline; Ipi, ipilimumab; Nivo, nivolumab; OT, one cycle of treatment; pCR, pathological complete response; pNR, pathological no response; npCR, near pathological complete response; pPR, pathological partial response; RNA-seq, RNA sequencing; Tira, tiragolumab; Tobe, tobemstomig

List of Independent Ethics Committees/Institutional Review Boards

EC/IRB Name and Address	IRB/EC Approval
Melanoma Institute Australia, Bellberry HREC, 123 Glen Osmond Road, Eastwood SA, 5063	16-Nov-2021
Hospital Universitario Vall d'Hebron, DÍAZ DE RADA PARDO, Calle Irunlarrea 3, Pamplona, 31008	27-Sep-2021
Institut Universitaire du Cancer de Toulouse - Oncopole, Central EC CPP, 59 Boulevard Pinel, Bron, 69500	19-Nov-2021
Gustave Roussy, Central EC CPP, 59 Boulevard Pinel, Bron, 69500	24-Nov-2021
Hôpital Saint-Louis, Central EC CPP, 59 Boulevard Pinel, Bron, 69500	19-Nov-2021
Azienda Ospedaliera Universitaria Senese, CET Comitato Etico Regione Toscana Area Vasta Sud, Strada delle Scotte n. 14, Siena, 53100<br>Azienda Ospedaliera Universitaria Senese, Comitato Etico Area Vasta Sud, Est, Viale Bracci, 16, Siena, 53100 (till 25-Aug-2023)	18-Oct-2021
European Institute of Oncology, CET Comitato Etico Regione Toscana Area Vasta Sud, Strada delle Scotte n. 14, Siena, 53100<br>European Institute of Oncology, Comitato Etico IRCCS IEO e CCM, Via Ripamonti 435, Milano, 20141<br>European Institute of Oncology, Comitato Etico Area Vasta Sud Est,Viale Bracci, 16 Siena 53100 (till 25-Aug-2023)	21-Jan-2022
Istituto Nazionale Tumori IRCCS "Fondazione G. Pascale", CET Comitato Etico Regione Toscana Area Vasta Sud, Strada delle Scotte n. 14, Siena, 53100<br>Istituto Nazionale Tumori IRCCS "Fondazione G. Pascale", Comitato Etico<br>Istituto Nazionale Tumori, Via Mariano Semmola, Napoli NA, 80131<br>Istituto Nazionale Tumori IRCCS "Fondazione G. Pascale", Comitato Etico Area Vasta Sud Est, Viale Bracci, 16, Siena, 53100 (till 25-Aug-2023)	11-Nov-2021
The University of Texas MD Anderson, Local IRB Office of Human Subjects Protection, 7007 Bertner Avenue - Unit 1637, Houston, TX, 77030	04-Jan-2022
City of Hope, Central IRB WCG IRB, 212 Carnegie Center, Suite 301, Princeton, 8540	27-Oct-2021
Moffitt Cancer Center, Local IRB Advarra IRB, 6100 Merriweather Dr, Suite 600, Columbia, MD, 77030	06-Oct-2021
Azienda Ospedaliera di Perugia - Ospedale Santa Maria della Misericordia, CET Comitato Etico Regione Toscana Area Vasta Sud, Strada delle Scotte n. 14, Siena, 53100<br>Azienda Ospedaliera di Perugia - Ospedale Santa Maria della Misericordia, Comitato Etico Area Vasta Sud Est, Viale Bracci, 16 , Siena, 53100 (till 25-Aug-2023)	16-Mar-2022
The Angeles Clinic and Research Institute, Central IRB WCG IRB, 212 Carnegie Center, Suite 301, Princeton, 8540	06-Apr-2022
CHU Hôpital de la Timone, Central EC CPP, 59 Boulevard Pinel, Bron, 69500	24-Nov-2021
